# The inclusion of de-oiled wet distillers grains in feedlot diets reduces the expression of lipogenic genes and fat content in Longissimus muscle from F1 Angus-Nellore cattle

**DOI:** 10.7717/peerj.7699

**Published:** 2019-10-28

**Authors:** Mateus S. Ferreira, Laís A. Tomaz, Maria B. Niehues, Márcio M. Ladeira, Rogério A. Curi, Luís A. Chardulo, Welder A. Baldassini, Cyntia L. Martins, Mário B. Arrigoni, Otávio R. Machado Neto

**Affiliations:** 1 Departamento de Produção Animal, Universidade Estadual Paulista, Botucatu, São Paulo, Brazil; 2 Departamento de Melhoramento e Nutrição Animal, Universidade Estadual Paulista, Botucatu, São Paulo, Brazil; 3 Departamento de Zootecnia, Universidade Federal de Lavras, Lavras, Minas Gerais, Brazil

**Keywords:** Chemical composition, Meat science, Nutrigenomics, Metabolism, Marbling, Meat science, Meat, Beef

## Abstract

The inclusion of agro-industry by-products originated from corn ethanol production has increased in animal nutrition in Brazil, reducing formulation costs. In the literature, there is no consensus on how the high inclusion of de-oiled wet distillers grains can affect beef quality and the expression of lipogenic genes in Longissimus muscle. The aim of this study was to evaluate the effects of WDG in the diet of F1 Angus-Nellore cattle on meat quality characteristics, chemical composition and expression of genes involved in lipid metabolism. A hundred F1 Angus-Nellore bulls, with average initial body weight (BW) of 369.5 ± 49 kg were used. The experiment was carried out in a randomized block design, and the animals were divided into two blocks (light and heavy) according to the initial body weight. The animals were fed diets containing levels of 0 (control), 15, 30 and 45% of WDG replacing dry corn and soybean meal. After 129 days of feedlot, the animals were slaughtered and samples of the *longissimus thoracis* (LT) muscle were collected for quality analyzes such as shear force (3, 10 and 17 aging days), color (luminosity, red, Chroma and Hue), cooking losses, pH and chemical composition (moisture, protein, lipids and ash contents). In addition, the expression of the *PPAR*α*, PPAR*γ*, SREBP-1c, SCD1, LPL, FABP4, FASN, ACOX, CPT2, GPX1* and *ACACA* genes was investigated in the LT muscle by real-time reverse transcription polymerase chain reaction (RT-PCR). Data were analyzed using polynomial contrasts (linear, quadratic and control vs. WDG). There was no interaction (*P* > 0.05) between aging times and the inclusion of WDG in the diets on the meat quality (pH, cooking losses, coloration and tenderness). However, diets with increasing levels of WDG caused a linear reduction (*P* = 0.01) in the intramuscular fat of LT. The lipogenic genes *SCD1, PPAR*γ*, FASN* and *CPT2* were less expressed (*P* < 0.05) in response to the inclusion of WDG. These results suggest that the inclusion of WDG reduced the expression of lipogenic genes and consequently the marbling of LT muscle without affecting tenderness (shear force) and meat color traits.

## Introduction

Currently, the production of corn ethanol in Brazil has increased along with the availability of by-products originated from this process in feedlot cattle diets. In countries such as the USA, distillers grains have been used in animal nutrition since the 1980s due to the high production of corn ethanol (Firkins, Berger & Fahey, 1985).

In the production of corn ethanol, after starch fermentation, the solid fraction of the remaining by-product tends to present the highest levels of protein (30%), fat (12%) and neutral detergent fiber (NDF = 36%), once the starch consumed in the alcoholic fermentation represents approximately 64% of the corn grain. However, ethanol plants have developed techniques to extract the oil from this by-product, making it rich in high digestibility fiber and rumen non-degradable protein (Jolly-Breithaupt et al., 2018).

Therefore, the replacement of ground dry corn by wet distillers grains leads to a reduction in starch intake, which may affect ruminal production of propionate, which is the main precursor of gluconeogenesis in ruminants. About 50–75% of the acetyl units used in intramuscular lipogenesis are derived from glucose, which might increase marbling in the meat (Smith & Crouse, 1984) when animals are fed diets rich in starch. It may explain the reduction in marbling score of the meat in animals fed WDG (Schoonmaker, Trenkle & Beitz, 2009).

The degree of marbling is one of the most important and definitive characteristics of meat quality since it is closely associated with the palatability of cooked meat (such as tenderness, juiciness and flavor) and influences consumer buying decisions (Smith et al., 1988). According to Picard et al. (2018), marbling is the most economically important and valuable trait for beef the industry and its improvement is a challenge for important markets such as the United States, Japan and Korea. The marbling score can be determined by visual evaluation by trained professionals using patterns of images on the muscular surface (Gerrard, Gao & Tan, 1996). However, visual score of marbling are subjective and may lead to underestimation or overestimation of scores, once some fat deposits (adipocytes) cannot be seen or quantified properly (Cheng et al., 2015; Gerrard, Gao & Tan, 1996). Thus, it is necessary to measure the amount of fat in the muscle and its relationship with the regulatory genes of the lipid metabolism.

Regarding meat tenderness, according to recent studies (Chao et al., 2018; Ribeiro et al., 2019), WDG-fed cattle may present lower shear force values (tender meat) when compared to animals fed ground corn. The literature suggests that this phenomenon is a consequence of the higher ingestion and deposition of unsaturated fatty acids (Ribeiro et al., 2018). According to these authors, when the animals were fed WDG with high fat content, there was a higher concentration of polyunsaturated fatty acids in the membrane of the sarcoplasmic reticulum, which may have anticipated the oxidation process of this membrane and the release of calcium in the muscle. However, more studies are necessary to prove this hypothesis.

In the literature, studies have been used high fat-WDG to evaluate carcass traits and beef quality (Buttrey et al., 2013; Hales, Cole & MacDonald, 2013). However, there are few studies evaluating the inclusion of de-oiled WDG on meat quality of feedlot cattle (Domenech-Pérez et al., 2017; Jolly-Breithaupt et al., 2018). The de-oiled-WDG used in the United States have about 9.2% fat (Jolly-Breithaupt et al., 2018), while industries in Brazil have generated this ingredient with fat content of less than 5%, which may justify the differences when compared to the results reported in the literature. In addition, most of the studies evaluated only the performance, carcass traits and beef quality of cattle (Buttrey et al., 2013; Hales et al., 2013; Domenech-Pérez et al., 2017). Few studies have investigated the molecular mechanisms that regulate these characteristics with WDG in the diet.

Therefore, the aim of this study was to evaluate the effects of the inclusion of increasing levels of de-oiled WDG on the chemical composition, expression of lipogenic genes in the Longissimus muscle and meat quality of feedlot cattle.

## Materials and Methods

All the procedures performed in the experiment were approved by the Ethics Committee on Animal Use (CEUA) of the School of Veterinary Medicine and Animal Science - UNESP (protocol no. 0067/2017).

### Facilities, management and experimental diets

The experiment was carried out at the School of Veterinary Medicine and Animal Science of the São Paulo State University, Botucatu, São Paulo, Brazil. A hundred F1 Angus-Nellore bulls with average initial body weight of 369.58 ± 49.17 kg were used. The animals were weighed at the beginning of the experiment, treated against endo- and ectoparasites and distributed in two blocks (denominated “light” and “heavy”) according to the initial body weight (iBW). Light animals had an iBW range from 290 to 367 kg (iBW mean = 336.61 ± 18.32 kg), whereas heavy animals had an iBW range from 368 to 495 kg (iBW mean = 415.87 ± 31.10 kg). The animals were allocated to collective covered pens with 5 animals each, with concrete floor cast, equipped with bunk and water trough. Then, the pens were randomly allocated among the four treatments, totaling 25 animals/treatment. The treatments consisted of increasing levels of wet distillers grains, being 0, 15, 30 and 45% inclusion in dietary DM (Table 1). All the WDG used in the experiment were produced by the same ethanol plant (SJC Bioenergia, Quirinópolis, Goiás, Brazil). The animals were fed for 129 days and the diets were given *ad libitum* twice a day (10 a.m. and 4 p.m.).

**Table 1:** Composition of the experimental diets.

Ingredients and diet composition	WDG (%)			
	0	15	30	45
Ingredients (% DM)				
*Tifton* 85	4.20	4.20	4.20	4.20
Sugarcane bagasse	7.10	7.10	7.10	7.10
Ground corn	74.92	65.27	52.00	38.73
Soybean meal	10.36	4.78	2.94	1.10
Wet distillers grains (WDG)	0	15.00	30.00	45.00
Supplement mineral-vitamin^a^	3.42	3.42	3.42	3.42
Potassium chloride	0	0.23	0.34	0.45
Nutritional composition (% DM)				
Dry matter (% NM)	86.78	78.08	69.40	60.72
Crude Protein	12.85	14.19	17.13	20.07
Ether Extract	3.42	3.53	3.54	3.55
Neutral Detergent Fiber	16.28	23.64	31.19	38.75
Non-fibrous carbohydrates	64.68	56.02	45.42	34.82
Starch	48.22	41.78	33.24	24.69
NEg (MJ/kg DM)	5.44	5.40	5.32	5.28

**Notes.**

a Supplement mineral-vitamin: 18.5% Ca; 1.9% S; 1.50% Mg; 4.3% Na; 1.60% P; 1714.00 ppm Zn; 1285 ppm Mn; 426.00 ppm Cu; 21 ppm I; 5.70 ppm Se; 8.5 ppm Co; 285.00 ppm Fe; 85700.00 UI Vit A; 11430.00 UI Vit D3; 128.00 UI Vit E; 32.5% Urea; 945.00 ppm of monensin sodium.

### Slaughter and collection of samples

The animals were slaughtered at the age 24 months at the end of the experimental period in a commercial slaughterhouse (Frigoestrela, Estrela D’Oeste, São Paulo, Brazil), where they were subjected to fasting for 24 h and free access to water. The animals were desensitized using the brain concussion technique with the aid of a captive dart gun, followed by bleeding, leather removal and evisceration. Subsequently, the carcasses were divided longitudinally and carcass samples (approximately 20 g) were collected from the *longissimus thoracis* (LT) muscle at the 12th rib in the left half, using RNA stabilizer solution (RNA*later*; Sigma-Aldrich, St. Louis, MO, United States) and stored in freezer at −80 °C until the beginning of molecular biology analyzes. In the refrigerator, the half-carcasses were refrigerated at 2–4 °C for 72 h. After cooling, four steaks (2.54 cm) were collected from the left half of the LT muscle for physical-chemical analyzes. The first steak was collected between the 12th and 13th ribs and the others from the cranial direction of the carcass.

### Beef aging and physical-chemical analyzes

The first three steaks were vacuum packed with polyethylene packages and kept in refrigerator at 2 °C for 3, 10 and 17 days, respectively. At the end of each aging period, the samples were frozen at −20 °C until the physical-chemical analyzes. Subsequently, samples were thawed at 4 °C for 24 h and exposed to oxygen for 30 min at 4 °C (blooming time). First, the meat pH was measured using a Hanna digital pH meter (Model HI 99163, Hanna Instruments, Woonsocket, RI) with penetration probe.

In the same steak, meat color (L * = luminosity, a * = red intensity, b * = yellow intensity) was measured using the CIELab system of the CR-400 colorimeter (light source A, absorbance angle 10, Y, 0.01 at 160.00% reflectance; Konica Minolta Sensing, Inc., Tokyo, Japan), following the procedures previously described by Baldassini et al. (2017). The colorimeter was calibrated using a standard black and white plate and then three color readings were performed on the surface of the LT muscle sample. An average of the three measurements was generated for each variable (L *, a * and b *). Chroma colorimetric indexes (color saturation) were calculated by the formula [(*a*∗)2 + (*b*∗)2]^0.5^ and the hue angle (H°) [*tang* − 1(*b*∗∕*a*∗)], as described by Cañeque et al. (2004).

The samples were placed in a grid over a glass refractory and weighed. Afterwards, a thermocouple was inserted into the geometric center of the samples, coupled to a digital thermometer model DT-612 (ATP Instrumentation, Ashby-de-la-Zouch, UK) to monitor the internal temperature of the samples. The steaks were roasted in a preheated oven (Feri90; Venâcio Aires, RS, Brazil) equipped with a thermostat to avoid temperature variation. When the internal temperature of the steak reached 40 °C the sample was turned and remained in the oven until reaching 71 °C internal temperature, according to the methodology described by Wheeler et al. (1994). Then, the samples were kept at room temperature for 15 min, weighed and refrigerated at 4 °C for 24 h. The cooking loss was determined by the weight difference before and after cooking. The cooking losses were measured from drip and evaporation losses. After cooling, eight cylinders with 1.27 cm diameter were removed from the parallel direction of the muscle fiber using a nozzle coupled to an industrial drill. The cylinders were sectioned in Salter Warner-Bratzler equipment with capacity of 25 kg and speed of 20 cm/minute. The results were presented in Newton (N) and eight replicate measurements per steak were performed to increase results accuracy.

Two animals per pen were randomly selected for chemical composition and gene expression analyzes, totaling 10 replicates per treatment. The analyzes of centesimal composition were performed in FoodScan™ (FOSS, Hillerød, Denmark). The samples were thawed at 4 °C for 24 h and the subcutaneous fat was removed from the LT muscle with the aid of a scalpel, then the steak was ground and homogenized for 5 min using a mixer, taking approximately 180 g of sample (Anderson, 2007). Three readings per sample were carried out. Samples were homogenized again and placed in the plate for the next reading. An average was obtained for the values of moisture, protein, fat and ash and the values were expressed as percentage.

### Gene expression

Gene expression tests were performed following procedures previously described (Teixeira et al., 2017). The design of target and reference primers was performed using sequences that are registered and published in the GenBank public data bank, a National Center for Biotechnology Information (NCBI) platform (Table 2). Primers were designed using OligoPerfect Designer software (Invitrogen, Karlsruhe, Germany) and synthesized (Invitrogen, Carlsbad, CA, USA). Total RNA was extracted from muscle samples using QIAzol (QIAGEN, Valencia, CA, USA) and treated with DNA-free DNase (Ambion, Austin, TX) according to the manufacturer’s instructions. To analyse the 28S and 18S rRNA bands, the total RNA was electrophoresed in a 1.0% (m/v) agarose gel, stained with GelRed nucleic acid gel stain (Biotium, Hayward, CA, USA) and visualized with a E-gel Imager Camera Hood (Life Technologies, Neve Yamin, Israel). The RNA quantity (ng/µL) and quality (260/280 and 260/230) were assessed using a spectrophotometer (DeNovix DS-11 Spectrophotometer) at 260 nm. cDNA synthesis was performed using the HighCapacity cDNA Reverse Transcription Kit (Applied Biosystems, Foster City, CA, USA) according to the manufacturer’s instructions, and samples were stored at −20 °C.

**Table 2:** Sequences (5′ to 3′) and efficiencies of the primers used in quantitative real-time PCR.

Symbol	Name	Forward (F) and reverse (R)	Access number	Amplicon (bp)	R^2^	Efficiency
*PPARα*	*Peroxisome proliferator-activated receptorα*	F CAATGGAGATGGTGGACACA	NM_001034036.1	95	0,992	99,2
		R TTGTAGGAAGTCTGCCGAGAG				
*PPARγ*	*Peroxisome proliferator-activated receptor gamma*	F CGACACAAACTGAACACACAGAGT	NM_181024.2	83	100	99,9
		R TCAGCGGGAAGGACTTTATG				
*SREBP-1c*	*Sterol regulatory element-binding protein-1c*	F GAGCCACCCTTCAACGAA	NM_001113302.1	88	0,985	94,6
		R TGTCTTCTATGTCGGTCAGCA				
*SCD1*	*Stearoyl-CoA desaturase 1*	F ACCATCACAGCACCTCCTTC	NM_173959.4	95	0,999	98
		R ATTTCAGGGCGGATGTCTTC				
*LPL*	*Lipoprotein lipase*	F CTCAGGACTCCCGAAGACAC	NM_001075120.1	98	0,99	96,7
		R GTTTTGCTGCTGTGGTTGAA				
*FABP4*	*Fatty acid binding protein 4*	F GGATGGAAAATCAACCACCA	NM_174314.2	73	0,995	97
		R GTGGCAGTGACACCATTCAT				
*FASN*	*Fatty acid synthase*	F ATCAACTCTGAGGGGCTGAA	U34794.1	83	0,974	99,5
		R CAACAAAACTGGTGCTCACG				
*ACOX*	*Acyl-coenzyme A oxidase 1*	F GCTGTCCTAAGGCGTTTGTG	BC102761.2	83	0,994	99
		R ATGATGCTCCCCTGAAGAAA				
*CPT2*	*Carnitine palmitoyltransferase 2*	F CTATTCCCAAACTTGAAGAC	NM_001045889.2	81	0,978	100
		R TTTTCCTGAACTGGCTGTCA				
GPX1	*Glutathione Peroxidase*	F GGAGATCCTGAATTGCCTGA	NM_174076.3	87	0,991	100
		R CCATTCACCTCGCACTTTTC				
ACACA	*Acetyl CoA carboxylase alfa*	F TGAAGAAGCAATGGATGAACACA	NM_174224.2	88	0,994	100
		R TTCAGACACGGAGCCAATAA				
*β*-*actin*	*β*-*Actin*	F GTCCACCTTCCAGCAGATGT	NM_173979.3	90	0,998	100
		R CAGTCCGCCTAGAAGCATTT				
*CASC3*	*Cancer susceptibility candidate 3*	F GGACCTCCACCTCAGTTCAA	NM_001098069.1	85	0,976	98
		R GTCTTTGCCGTTGTGATGAA				

Real-time qPCR (RT-qPCR) was performed on an Eppendorf Realplex system (Eppendorf, Hamburg, Germany) with a SYBR Green detection system (Applied Biosystems, Foster City, CA, USA). PCRs were incubated in a 96-well plate at 50 C for 2 min, followed by 95 °C for 10 min, followed by 40 cycles of 95 °C for 15 s and 60 °C for 1 min. The RT-qPCR analyses of each studied gene were performed using cDNA from biological replicates, with two technical replicates per biological replicate. Four reference genes were tested, and the best individual gene or combination of endogenous controls was chosen using the web-based tool RefFinder, which selected *β*-actin and CASC3 genes as more stable for use in muscle gene expression. A validation assay was performed to demonstrate that the amplification efficiencies of the target and reference genes were approximately equivalent. Standard curves were generated for the studied genes with the following dilutions: 1:5, 1:25, 1:125, 1:625 and 1:3125. Relative expression levels were calculated according to the method described by Pfaffl (2001), which is based on Ct values that are corrected for the amplification efficiency of each primer pair.

### Removal of animals and samples

During the experiment, three animals were taken from the study because they presented symptoms of polioencephalomalacia (*n* = 2) and had their urethras ruptured due to the practice of sodomy (*n* = 1). During the storage of gene expression samples, four samples had their identifications lost, two from the control group and two from the animals that received 30% WDG. Therefore, eight, 10, eight and 10 samples were used to analyze chemical composition and gene expression for the 0, 15, 30 and 45% treatments, respectively. As the objective of the experiment was to analyze chemical composition and gene expression from the same animals, the chemical composition of the lost samples was not analyzed either.

### Statistical analyzes

Data were tested for normality through the Shapiro–Wilk Test, PROC UNIVARIATE procedure of SAS 9.4 (SAS Institute, Cary, NC, USA, 2011). The genes SCD1, PPAR*γ*, SREBP-1c, ACOX, FASN, CPT2, FABP4, GPX1, LPL, PPAR*α* and ACACA did not present normal distribution and were transformed using PROC RANK (SAS 9.4).

The chemical composition and gene expression were analyzed using polynomial (linear, quadratic and control vs. WDG) contrasts using the PROC MIXED procedure, in which the inclusion of WDG was considered as fixed effect, the block and the pen as random effect, according to the model:

\begin{eqnarray*}& & Yijk=\mathrm{\mu }+Bi+Aj+Wk+eijk \end{eqnarray*}

where: µ= overall mean, *Bi* = random effect of block, *Aj* = random effect of pen, Wk = WDG levels effect (0, 15, 30 e 45% DM basis) and eijl = experimental error.

Meat quality data were submitted to Tukey’s test using the PROC MIXED procedure, in which the model included the WDG level, aging days and WDG level × days of aging as fixed effect, block and pen as random effect, according to the model:

\begin{eqnarray*}& & Yijkl=\mathrm{\mu }+Bi+Aj+Wk+Dl+WDkl+eijkl \end{eqnarray*}

where: µ= overall mean, *Bi* = random effect of block, *Aj* = random effect of pen, *Wk* = WDG levels effect (0, 15, 30 e 45% DM basis), *Dl* = efeito dos dias de maturação, *WDkl* = interaction between the level of WDG and aging day and eijkl = experimental error.

In both models, animals were considered as the experimental unit. Significance was considered when *P* < 0.05.

## Results

There was no significant difference (*P* > 0.05) for moisture, protein and ash in LT muscle regardless of the level of WDG. However, there was a reduction (*P* < 0.05) in the LT muscle fat content in all treatments (Table 3). On average, this reduction was 15.73% when the animals consumed WDG. It was also observed by polynomial contrasts that the inclusion of WDG reduced linearly (*P* = 0.01) the intramuscular fat content in LT. Then, the lowest intramuscular fat content was observed in the treatment with 45% de-oiled WDG.

**Table 3:** Chemical composition of the *longissimus thoracis* muscle of F1 Angus-Nellore cattle fed different levels of de-oiled wet distillers grains (WDG).

Item (%)	WDG (% DM)				SEM^a^	*P-value*^b^		
	0	15	30	45		C vs W	L	Q
Moisture	74.41	74.34	74.11	74.49	0.27	0.71	0.99	0.31
Protein	23.20	23.22	23.05	23.58	0.20	0.69	0.24	0.18
Fat	2.67	2.32	2.40	2.03	0.16	0.03	0.01	0.94
Ash	1.14	1.09	1.10	1.12	0.02	0.15	0.62	0.09

**Notes.**

a Standard Error Mean.

b C vs W, control vs WDG; L, linear effect and Q, quadratic effect.

**Figure 1:**
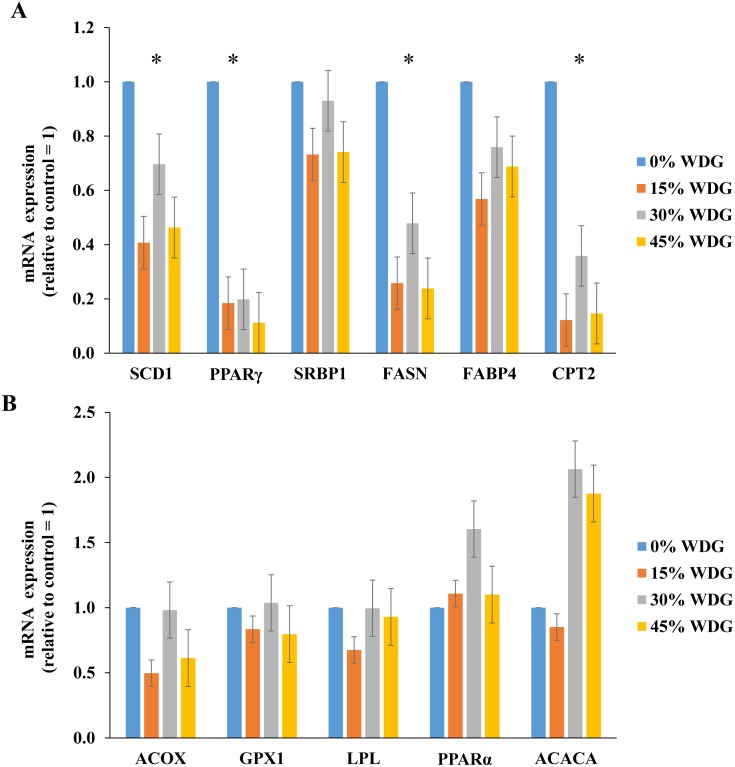
Expression of lipogenic genes in the *longissimus thoracis* muscle of F1 Angus-Nellore finished in feedlot fed different levels of wet distillers grains (WDG). (*) Statistical effect (*P* < 0.05) between treatments. (A) Genes: Sterol regulatory element-binding protein-1c (SCD1), Peroxisome proliferator-activated receptor gamma (PPAR*γ*), Stearoyl-CoA desaturase 1 (SRBP1), Fatty acid synthase (FASN), Fatty acid binding protein 4 (FABP4), Carnitine palmitoyltransferase 2 (CPT2); (B) Genes: Acyl-coenzyme A oxidase 1 (ACOX), Glutathione Peroxidase (GPX), Lipoprotein lipase (LPL), Peroxisome proliferator-activated receptor *α* (PPAR*α*), and Acetyl CoA carboxylase alfa (ACACA).

The inclusion of WDG in the diets resulted in lower expression of lipogenic genes PPAR*γ*, SCD, FAS and CPT2 (*P* < 0.05). On average, there was a reduction of 83.5%, 79.1%, 67.5% and 47.8% in the expression of the PPAR*γ*, CPT2, SCD1 and FASN genes, respectively (Fig. 1). PPARy gene expression reduced linearly with the increase in WDG levels (*P* < 0.01). Similar results were observed (Fig. 1A) on SCD, FAS and CPT2 genes (*P* < 0.05).

The inclusion of WDG reduced the expression of lipogenic genes and, consequently, the amount of intramuscular fat, confirming this study’s hypothesis. However, there was no effect (*P* > 0.05) of WDG inclusion levels on meat quality traits such as pH, color (Table 4), cooking loss and tenderness (shear force) measured at three aging times (Table 5). In addition, there was no interaction (*P* > 0.05) between the level of WDG in the diets and aging time. In the present study, these results indicated that the inclusion of WDG in the diet did not affect meat tenderness.

**Figure 2:**
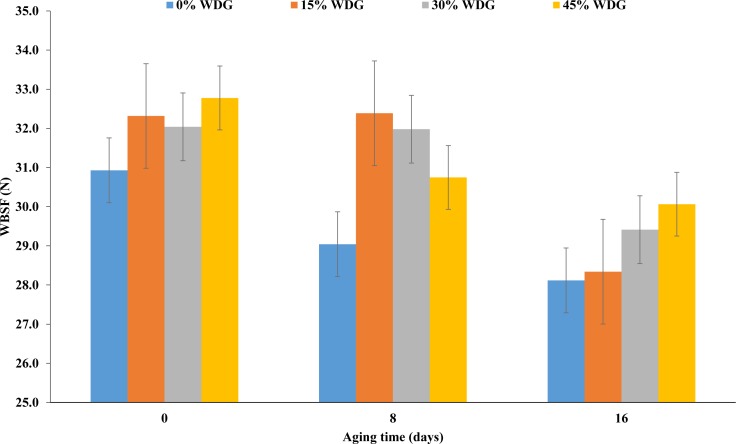
Shear force (WBSF) of the *longissimus thoracis* muscle at three aging times (0, 8 and 16 postmortem days) of F1 Angus-Nellore (*n* = 100) finished in feedlot fed different levels of de-oiled wet distillers grains (WDG).

The steaks submitted to aging showed lower pH than the ones that were not (*P* < 0.01). Regardless of the treatment, the average final pH was 5.83, 5.70 and 5.73 for aging days 0, 8 and 16, respectively. The luminosity of samples at aging 16 was 3% higher than in samples at aging 0 (*P* < 0.01). However, there was a lower cooking loss when the meat underwent aging (*P* < 0.05). Also, there was a reduction in the shear force in the steaks submitted to aging (*P* < 0.01). On average, there was a reduction of 3 N at 16 days of aging regardless of the treatment (Fig. 2).

## Discussion

### Diet effects on intramuscular fat

The hypothesis of the present study was that the inclusion of WDG with low fat content in finishing cattle diets reduces the expression of genes involved in the lipid metabolism of LT muscle and, therefore, affects the deposition of intramuscular fat. The results confirm this hypothesis. In other studies, although applying an intramuscular fat score methodology (Schoonmaker, Trenkle & Beitz, 2010) according to the USDA quality and yield grades, researchers reported reduced marbling in the meat of Angus cattle finished in feedlot with diets containing 40% WDG. In this study, a proportional reduction of 18% in the marbling score (control diet versus 40% WDG) occurred, corroborating the reduction of 23.97% observed in the present study on the meat lipid content, when the animals consumed diets with 45% WDG. In addition, the reduction in intramuscular fat content is not a result of the lower weight gain of the animals, since the inclusion of the by-product promoted better performance (Ferreira et al., 2018). It is possible to infer that the change in ruminal fermentation pattern due to the change in the nutritional profile of the diet (reduction of starch, especially) was responsible for the reduction in intramuscular fat content.

**Table 4:** Color and pH characteristics of the *longissimus thoracis* muscle at three aging times (3, 10 and 17 postmortem days) of F1 Angus-Nellore cattle (*n* = 100) fed different levels of de-oiled wet distillers grains (WDG).

Item^3^	Aging time (days)												SEM^1^	*P-value*^2^		
WDG (%DM)	3				10				17					WDG	Day	W × D
	0	15	30	45	0	15	30	45	0	15	30	45				
pH	5.81a	5.84a	5.88a	5.77a	5.73bc	5.68bc	5.74bc	5.66bc	5.73c	5.72c	5.74c	5.71c	0.04	0.14	<.01	0.84
L*	37.96a	37.56a	38.11a	37.20a	37.77a	37.31a	36.70a	37.51a	39.15b	38.63b	38.04b	39.28b	0.76	0.87	<.01	0.70
a*	20.30	20.05	20.98	20.48	19.78	20.21	19.91	19.09	20.34	20.12	20.47	20.89	0.56	0.92	0.06	0.48
b*	9.60	9.60	9.85	9.65	9.22	9.54	9.26	9.03	9.10	9.27	9.38	9.48	0.36	0.95	0.08	0.88
Chroma	22.48	22.24	23.41	22.66	21.96	22.57	21.93	21.42	22.42	22.29	23.01	23.37	0.60	0.85	0.05	0.36
Hue	25.26a	25.66a	24.79a	25.05a	24.80ab	25.07ab	24.63ab	24.47ab	23.89b	24.36b	24.37b	24.11b	0.52	0.68	0.01	0.95

**Notes.**

1 Standard error mean.

2 WDG, Effect of the levels of WDG; Day, effect of the aging day; W × D, interaction between WDG and Day.

3 L*, lightness; a*, redness; b*, yellowness; Chroma, chromaticity or quantity of color; Hue, real color.

**Table 5:** Cooking loss and shear force of the longissimus thoracis muscle at three aging times (3, 10 and 17 postmortem days) of F1 Angus-Nellore cattle (*n* = 100) fed de-oiled wet distillers grains (WDG).

Item^3^	Aging time (days)												SEM^1^	*P-value*^2^		
WDG (%DM)	3				10				17					WDG	Day	W × D
	0	15	30	45	0	15	30	45	0	15	30	45				
Evp, %	17.68	17.34	17.02	18.57	17.25	18.33	16.36	17.87	17.56	16.99	16.83	16.78	0.68	0.33	0.27	0.31
DL, %	2.52a	2.80a	2.54a	2.33a	2.00bc	2.29bc	2.25bc	2.15bc	1.98c	2.10c	2.13c	2.03c	0.17	0.24	<.01	0.83
CL, %	20.09a	20.15a	19.69a	21.06a	19.85ab	20.58ab	19.24ab	19.89ab	19.74b	19.18b	18.72b	18.75b	0.74	0.47	0.03	0.73
WBSF, N	30.92a	32.31a	32.04a	32.77a	29.04a	32.38a	31.98a	30.74a	28.11b	28.34b	29.41b	30.06b	1.24	0.23	<.01	0.86

**Notes.**

1 Standard error mean.

2 WDG, Effect of the levels of WDG.

3 Evp, Steak Evaporation Loss; DL, Steak Drip Loss; CL, Cooking Loss; WBSF, Warner Blatzler Shear Force.

The inclusion of WDG replacing dry corn reduced the starch content of the experimental diets (from 47.63% to 24.63%). When starch is fermented in the rumen, there is a higher production of ruminal propionate, which is the main precursor of hepatic gluconeogenesis in ruminants (Bauman, Mellenberger & Derrig, 1973). According to the same authors, both rumen acetate and glucose synthesized from propionate are the main substrates that form acetyl units in de novo intramuscular synthesis. However, from 50 to 75% of the acetyl-CoA units formed are derived from blood glucose, and ruminal propionate seems to have a direct influence in this process (Smith & Crouse, 1984). The incorporation of acetate into fatty acid formation seems to be greater in subcutaneous adipose tissue than in muscle tissue, and glucose seems to have an inverse effect, being more incorporated into intramuscular tissue when compared to the subcutaneous (Rhoades et al., 2007). These results help to explain the reduction in intramuscular fat when WDG was included in the diets to replace dry corn. However, there are contradictory theories about the participation of glucose as a precursor of the acetyl units in the intramuscular fat in cattle (Nayananjalie et al., 2015).

After the formation of acetyl-CoA units, there is a carboxylation of this molecule that originates malonyl-CoA, through the enzyme acetyl-CoA carboxylase. Then acetyl-CoA and malonyl-CoA molecules bind through multiple enzymatic reactions originating long chain fatty acids, through the enzyme fatty acid synthase, encoded by the FASN gene (Ladeira et al., 2016). In other words, the inclusion of WDG in the diets linearly reduced FASN gene expression (*P* < 0.01), potentially leading to a lower synthesis of the enzyme fatty acid synthase and lower intramuscular lipogenesis.

### Nutrients and lipogenic genes expression

However, there are transcription factors of the PPAR family that regulate the expression of some genes responsible for lipogenesis, with a major role in the control of glucose and lipid regulation (Liu et al., 2014). Among these transcription factors, PPAR*α* seems to activate the genes that are responsible for the oxidation of fatty acids, whereas PPAR*γ* has an antagonistic effect, activating lipogenic and adipogenic genes, being more expressed in obese individuals (Gervois et al., 2000). PPAR*γ* can be regulated by blood glucose concentrations, and it is more expressed when glucose is in high amounts (Liu et al., 2014). This may explain what happened in the present study since the inclusion of WDG in diets may have reduced the amount of blood glucose synthesized through ruminal propionate, reducing the expression of genes that encode PPAR*γ*. Fatty acid synthase has a direct action on PPAR*γ* regulation and adipogenesis, and the lower the synthesis of this enzyme, the lower the expression of PPAR*γ* and, as a consequence, there is a reduction in adipose tissue (Lodhi et al., 2012), which may also have helped in the lower expression of PPAR*γ*.

The transcription factor PPAR*γ* may also play a role in the genes that stimulate fatty acid oxidation, with the CPT2 gene, the gene encoding carnitine palmitotransferase, showing that as PPAR*γ* is silenced, there is a reduction in expression of CPT2 (Sharma et al., 2012). A similar effect of gene expression was observed in the present study, once PPAR*γ* expression was linearly reduced, this effect was also found for the CPT2 gene (*P* < 0.01).

The inclusion of WDG in the diets reduced linearly SCD1 gene expression (*P* < 0.01). This effect may also be associated with the reduction in starch of the diets (Table 1). SCD1 encodes the enzyme stearoyl-CoA desaturase and its main function is the desaturation of fatty acids, transforming saturated into unsaturated (SFA in MUFA in meat). This gene is more expressed in diets with large amounts of starch as it provides higher meat marbling due to *de novo* synthesis (Graugnard et al., 2009)*.* Therefore, as the ingestion impacts the expression of some key lipogenic genes, such as FASN, this effect reflects on other genes that are important to lipogenesis, such as PPAR*γ*, SCD1 and CPT2, drastically reducing muscle fat and meat marbling. Moreover, Ceciliani et al. (2018) reported that the integration of data derived from gene expression analyses with those from protein expression studies should be considered to draw a complete and reliable picture of the functions and the activities of adipose tissue due its fundamental importance for the quality of carcasses. Thus, in further nutrigenomic studies protein expression assays should be performed to integrate with data from fat gene expression, dissecting the molecular mechanism involved in intramuscular fat deposition.

### De-oiled WDG and beef quality

According to Ribeiro et al. (2018) when animals are fed WDG with a high fat content, there is a higher concentration of polyunsaturated fatty acids in the membrane of the sarcoplasmic reticulum, anticipating the oxidation process of this membrane and the release of calcium in the muscle. These events may accelerate the onset of post-mortem proteolysis, reducing meat toughness. In other words, the inclusion of WDG in animal diets does not affect the tenderness (shear force) during the aging process (Table 5). Considering that plants tend to extract fat from the by-product, their inclusion in the diets aiming at increasing meat tenderness should not become a common practice in quality meat production systems. Our results suggest that the inclusion of WDG reduced the expression of lipogenic genes and consequently, the marbling of LT muscle, without impairing the characteristics of tenderness (shear force) and meat color. The molecular mechanism associated with marbling and tenderness traits is often investigated in the literature, as reviewed by Picard, Gagaoua & Hollung (2017). According to researchers, molecular biology studies using both proteomics and gene expression approaches led to a substantial increase in knowledge in meat science, confirming the important role of energy metabolism and proteolysis in muscle to meat conversion. Thus, the gene expression assay performed in the present study was also interesting for understanding the mechanisms involved in tenderness (e.g., shear force).

Several studies have already shown improvement in meat tenderness with increasing aging time (Sañudo et al., 2004; Monsón, Sañudo & Sierra, 2005; Cho et al., 2016; Chao et al., 2018). In addition, some studies involving beef aging time have already shown an increase in luminosity (L *) and lower cooking loss with increasing aging time in the LT muscle (Vitale et al., 2014; Cho et al., 2016), corroborating the results obtained in the present study.

Marbling is a trait of great economic importance because, as well as color, it can influence the consumer’s purchasing decision. According to Wheeler, Cundiff & Koch (1994), marbling would explain at most 5% of the variability observed in meat palatability. However, according to this study, there is a large variation in tenderness within each class of marbling score in *Bos taurus* and *Bos indicus* animals. This means that it is possible to find meat that is considered tough or tender with different degrees of intramuscular fat. In Zebu animals, for example, researchers found a negative correlation between shear force and marbling score (*r* =  − 0.18; *P* < 0.001) but found no association between shear force and chemically-extracted intramuscular fat (*r* =  − 0.01) (Baldassini et al., 2017). Although there was a significant reduction in intramuscular fat content in the present study, the magnitude of this change was not enough to influence meat tenderness by shear force. This was probably because the animals used in this study are non-castrated males, which tend to present meat with low intramuscular fat.

## Conclusion

The inclusion of de-oiled WDG promotes significant reduction in meat intramuscular fat content without affecting tenderness. The reduction in intramuscular fat content is a consequence of the lower expression of lipogenic genes. The use of WDG in high proportions and for extended periods, as in this study, might be a problem to obtain high degrees of marbling.

Considering that marbling is not solely determined by gene expression changes, translational regulation and post-translational modifications play important roles in the determination of phenotype. For future studies, perhaps the use of techniques such as proteomics would help in understanding how beef quality may be affected by these nutritional treatments.

##  Supplemental Information

10.7717/peerj.7699/supp-1 Data S1 Raw Data: Chemical Composition

10.7717/peerj.7699/supp-2 Data S2 Raw Data: meat quality for data analyses

10.7717/peerj.7699/supp-3 Data S3 Raw Data: gene expression for data analyses

## Additional Information and Declarations

### Data Availability

The following information was supplied regarding data availability: The raw measurements are available in the Supplemental Files.
