# Supplementary material for: The inclusion of de-oiled wet distillers grains in feedlot diets reduces the expression of lipogenic genes and fat content in Longissimus muscle from F1 Angus-Nellore cattle
Source: PeerJ. 2019 Oct 28;7:e7699. doi: 10.7717/peerj.7699 (PMC6822641; doi:10.7717/peerj.7699)
Supplement: Data S1 [file peerj-07-7699-s001.docx]

| Animal | Block | Treatment | Pen | Fat | Protein | Moisture | Ash |
| --- | --- | --- | --- | --- | --- | --- | --- |
| 1 | 2 | 15 | 23 | 1.79 | 23.09 | 74.03 | 1.09 |
| 6 | 2 | 15 | 15 | 2.91 | 23.72 | 72.33 | 1.05 |
| 7 | 1 | 15 | 7 | 2.24 | 23.09 | 73.58 | 1.08 |
| 9 | 1 | 15 | 7 | 3.29 | 22.26 | 73.34 | 1.11 |
| 10 | 1 | 15 | 7 | 2.02 | 23.34 | 73.63 | 1.00 |
| 12 | 1 | 15 | 18 | 1.83 | 22.46 | 74.57 | 1.15 |
| 13 | 2 | 15 | 15 | 3.19 | 22.95 | 72.81 | 1.04 |
| 20 | 2 | 15 | 23 | 2.42 | 23.42 | 73.10 | 1.05 |
| 23 | 2 | 15 | 15 | 1.98 | 23.13 | 73.73 | 1.16 |
| 24 | 1 | 15 | 7 | 1.31 | 22.68 | 74.85 | 1.16 |
| 25 | 1 | 45 | 9 | 1.79 | 23.04 | 74.11 | 1.06 |
| 31 | 1 | 45 | 9 | 1.92 | 23.26 | 73.67 | 1.15 |
| 32 | 1 | 45 | 12 | 2.06 | 22.70 | 74.11 | 1.13 |
| 33 | 1 | 45 | 16 | 1.89 | 22.81 | 74.16 | 1.14 |
| 35 | 2 | 45 | 17 | 2.54 | 23.19 | 73.15 | 1.12 |
| 37 | 1 | 45 | 16 | 1.98 | 23.48 | 73.44 | 1.10 |
| 41 | 2 | 45 | 17 | 1.59 | 23.95 | 73.37 | 1.10 |
| 46 | 2 | 45 | 17 | 1.85 | 24.07 | 72.91 | 1.17 |
| 47 | 1 | 45 | 12 | 2.00 | 22.99 | 73.89 | 1.12 |
| 48 | 2 | 45 | 26 | 2.44 | 23.45 | 72.99 | 1.12 |
| 55 | 2 | 30 | 25 | 2.25 | 23.34 | 73.27 | 1.14 |
| 60 | 1 | 30 | 13 | 2.21 | 23.30 | 73.41 | 1.08 |
| 61 | 2 | 30 | 21 | 2.11 | 22.02 | 74.71 | 1.16 |
| 62 | 2 | 30 | 25 | 2.74 | 22.89 | 73.37 | 1.00 |
| 67 | 1 | 30 | 13 | 2.63 | 22.07 | 74.20 | 1.11 |
| 70 | 1 | 30 | 20 | 1.95 | 23.26 | 73.74 | 1.05 |
| 71 | 1 | 30 | 10 | 2.94 | 23.03 | 72.96 | 1.07 |
| 73 | 2 | 30 | 21 | 2.27 | 23.40 | 73.12 | 1.21 |
| 78 | 1 | 0 | 11 | 2.54 | 23.87 | 72.45 | 1.14 |
| 79 | 1 | 0 | 8 | 2.62 | 22.64 | 73.68 | 1.05 |
| 82 | 1 | 0 | 22 | 3.05 | 22.59 | 73.29 | 1.07 |
| 84 | 2 | 0 | 19 | 2.21 | 23.26 | 73.38 | 1.14 |
| 92 | 1 | 0 | 8 | 2.21 | 22.80 | 73.94 | 1.05 |
| 95 | 2 | 0 | 24 | 2.30 | 23.26 | 73.11 | 1.33 |
| 96 | 1 | 0 | 11 | 3.24 | 21.68 | 73.84 | 1.25 |
| 99 | 2 | 0 | 24 | 2.85 | 22.84 | 73.25 | 1.07 |
