# Supplementary material for: The inclusion of de-oiled wet distillers grains in feedlot diets reduces the expression of lipogenic genes and fat content in Longissimus muscle from F1 Angus-Nellore cattle
Source: PeerJ. 2019 Oct 28;7:e7699. doi: 10.7717/peerj.7699 (PMC6822641; doi:10.7717/peerj.7699)
Supplement: Data S2 [file peerj-07-7699-s002.docx]

| Animal | Pen | Block | Treatment | Aging | pH | Volatile loss | Drip loss | Cooking loss | L* | a* | b* | Chroma | Hue | WBSF |
| --- | --- | --- | --- | --- | --- | --- | --- | --- | --- | --- | --- | --- | --- | --- |
| 1 | 23 | 4 | 15 | 0 | 5.850 | 18.661 | 2.854 | 21.515 | 35.993 | 20.680 | 9.837 | 22.900 | 0.444 | 36.959 |
| 2 | 14 | 1 | 15 | 0 | 5.870 | 17.531 | 2.351 | 19.882 | 38.063 | 20.063 | 9.393 | 22.153 | 0.438 | 28.501 |
| 3 | 18 | 2 | 15 | 0 | 5.720 | 17.298 | 2.688 | 19.985 | 38.393 | 23.650 | 11.530 | 26.311 | 0.454 | 25.742 |
| 4 | 18 | 2 | 15 | 0 | 5.790 | 23.385 | 4.132 | 27.517 | 35.680 | 20.570 | 9.150 | 22.513 | 0.419 | 40.759 |
| 5 | 15 | 5 | 15 | 0 | 5.900 | 16.696 | 2.923 | 19.619 | 38.767 | 23.797 | 10.203 | 25.892 | 0.405 | 25.988 |
| 6 | 15 | 5 | 15 | 0 | 5.880 | 16.449 | 2.552 | 19.001 | 37.533 | 22.793 | 10.987 | 25.303 | 0.449 | 33.465 |
| 7 | 7 | 3 | 15 | 0 | 5.900 | 14.506 | 1.960 | 16.466 | 36.613 | 19.897 | 9.490 | 22.044 | 0.445 | 21.636 |
| 8 | 15 | 5 | 15 | 0 | 5.910 | 18.737 | 1.864 | 20.601 | 34.783 | 22.827 | 11.070 | 25.369 | 0.452 | 36.468 |
| 9 | 7 | 3 | 15 | 0 | 5.640 | 21.128 | 4.397 | 25.525 | 40.753 | 20.983 | 9.337 | 22.967 | 0.419 | 36.223 |
| 10 | 7 | 3 | 15 | 0 | . | 16.333 | 2.687 | 19.020 | 38.993 | 23.833 | 12.287 | 26.814 | 0.476 | 34.323 |
| 11 | 18 | 2 | 15 | 0 | . | 17.924 | 3.230 | 21.153 | 36.083 | 20.750 | 10.203 | 23.123 | 0.457 | 29.420 |
| 12 | 18 | 2 | 15 | 0 | 6.180 | 14.887 | 2.274 | 17.161 | 32.260 | 15.687 | 6.367 | 16.929 | 0.386 | 40.330 |
| 13 | 15 | 5 | 15 | 0 | 5.940 | 17.913 | 2.624 | 20.537 | 38.217 | 20.580 | 9.477 | 22.657 | 0.432 | 35.549 |
| 15 | 18 | 2 | 15 | 0 | 5.850 | 19.426 | 2.417 | 21.843 | 33.830 | 16.737 | 7.380 | 18.292 | 0.415 | 37.265 |
| 16 | 14 | 1 | 15 | 0 | 5.830 | 23.196 | 2.689 | 25.884 | 36.760 | 16.549 | 8.953 | 18.816 | 0.496 | 41.065 |
| 17 | 7 | 3 | 15 | 0 | 6.130 | 17.961 | 2.997 | 20.958 | 36.070 | 15.157 | 8.747 | 17.499 | 0.523 | 39.043 |
| 18 | 23 | 4 | 15 | 0 | 5.420 | 15.617 | 3.205 | 18.822 | 31.507 | 15.963 | 6.477 | 17.227 | 0.385 | . |
| 19 | 14 | 1 | 15 | 0 | . | 13.519 | 2.370 | 15.889 | 41.127 | 15.177 | 10.227 | 18.301 | 0.593 | 25.865 |
| 20 | 23 | 4 | 15 | 0 | 6.080 | 12.253 | 2.495 | 14.749 | 40.847 | 21.867 | 10.687 | 24.338 | 0.455 | 28.807 |
| 21 | 14 | 1 | 15 | 0 | . | 14.570 | 3.981 | 18.552 | 39.240 | 20.227 | 9.643 | 22.408 | 0.445 | 25.804 |
| 22 | 14 | 1 | 15 | 0 | 5.640 | 15.939 | 2.491 | 18.430 | 42.457 | 21.203 | 10.257 | 23.554 | 0.451 | 26.539 |
| 23 | 15 | 5 | 15 | 0 | . | 17.496 | 3.696 | 21.192 | 43.280 | 22.057 | 11.023 | 24.658 | 0.463 | 29.726 |
| 24 | 7 | 3 | 15 | 0 | 5.630 | 18.103 | 1.394 | 19.497 | 37.737 | 20.103 | 8.750 | 21.925 | 0.411 | 31.259 |
| 25 | 9 | 3 | 45 | 0 | 6.090 | 20.331 | 2.203 | 22.534 | 31.577 | 17.243 | 7.347 | 18.743 | 0.403 | 36.468 |
| 26 | 26 | 4 | 45 | 0 | 5.780 | 14.700 | 1.689 | 16.389 | 40.303 | 23.537 | 11.167 | 26.051 | 0.443 | 26.294 |
| 27 | 12 | 1 | 45 | 0 | . | 18.065 | . | 23.570 | 37.663 | 18.093 | 7.653 | 19.645 | 0.400 | 34.691 |
| 28 | 17 | 5 | 45 | 0 | 5.560 | 19.142 | 1.783 | 20.925 | 38.183 | 21.683 | 9.537 | 23.688 | 0.414 | 26.233 |
| 29 | 26 | 4 | 45 | 0 | 5.980 | 18.844 | 2.374 | 21.218 | 39.853 | 21.577 | 10.277 | 23.899 | 0.444 | 51.301 |
| 30 | 26 | 4 | 45 | 0 | 5.910 | 16.429 | 3.371 | 19.800 | . | 22.877 | 12.013 | 25.839 | 0.484 | 26.723 |
| 31 | 9 | 3 | 45 | 0 | 5.660 | 24.315 | 3.320 | 27.635 | 32.433 | 19.273 | 10.520 | 21.957 | 0.500 | . |
| 32 | 12 | 1 | 45 | 0 | . | 14.621 | 3.370 | 17.991 | 31.500 | 16.080 | 6.223 | 17.242 | 0.369 | 42.291 |
| 33 | 16 | 2 | 45 | 0 | 6.290 | 25.240 | 2.371 | 27.611 | 32.500 | 16.777 | 6.013 | 17.822 | 0.344 | 42.414 |
| 34 | 12 | 1 | 45 | 0 | 5.640 | 19.863 | 1.336 | 21.198 | 35.423 | 21.277 | 9.547 | 23.320 | 0.422 | 34.752 |
| 35 | 17 | 5 | 45 | 0 | 5.890 | 20.085 | 1.800 | 21.884 | 38.433 | 18.070 | 7.870 | 19.709 | 0.411 | 29.910 |
| 36 | 26 | 4 | 45 | 0 | . | 15.673 | 1.889 | 17.561 | 33.210 | 19.627 | 8.653 | 21.450 | 0.415 | 25.559 |
| 37 | 16 | 2 | 45 | 0 | 5.550 | . | 1.287 | . | 39.430 | 24.657 | 11.833 | 27.349 | 0.447 | 28.930 |
| 38 | 16 | 2 | 45 | 0 | 5.680 | 18.052 | 2.229 | 20.281 | 40.640 | 22.590 | 10.647 | 24.973 | 0.440 | 19.307 |
| 39 | 9 | 3 | 45 | 0 | 5.570 | 21.191 | 3.017 | 24.208 | 36.310 | 21.663 | 9.973 | 23.849 | 0.431 | 38.369 |
| 40 | 12 | 1 | 45 | 0 | 6.100 | 16.778 | 2.270 | 19.048 | 39.120 | 18.630 | 8.157 | 20.337 | 0.413 | 38.614 |
| 41 | 17 | 5 | 45 | 0 | 5.590 | 19.818 | 3.026 | 22.844 | 37.963 | 23.137 | 11.680 | 25.918 | 0.468 | 32.362 |
| 42 | 17 | 5 | 45 | 0 | 5.500 | 17.515 | 2.344 | 19.858 | 34.563 | 15.597 | 9.020 | 18.017 | 0.524 | 42.291 |
| 43 | 16 | 2 | 45 | 0 | 5.570 | 24.542 | 3.796 | 28.338 | 37.027 | 23.840 | 11.800 | 26.600 | 0.460 | 47.501 |
| 44 | 9 | 3 | 45 | 0 | 5.870 | 16.401 | 2.198 | 18.599 | 39.610 | 21.023 | 11.323 | 23.879 | 0.494 | 19.736 |
| 45 | 9 | 3 | 45 | 0 | . | 16.363 | 3.073 | 19.436 | 39.863 | 21.247 | 10.320 | 23.620 | 0.452 | 26.110 |
| 46 | 17 | 5 | 45 | 0 | 6.030 | 14.756 | 1.488 | 16.244 | 37.793 | 19.840 | 9.307 | 21.914 | 0.439 | 30.584 |
| 47 | 12 | 1 | 45 | 0 | 5.660 | 17.624 | 2.872 | 20.496 | 42.160 | 22.150 | 10.807 | 24.646 | 0.454 | 27.030 |
| 48 | 26 | 4 | 45 | 0 | 5.420 | 18.745 | 1.354 | 20.099 | 39.853 | 21.517 | 9.893 | 23.682 | 0.431 | 30.830 |
| 49 | 16 | 2 | 45 | 0 | . | 16.126 | 1.367 | 17.493 | . | . | . | . | . | 28.439 |
| 50 | 21 | 5 | 30 | 0 | 5.990 | 14.186 | 1.583 | 15.769 | 38.550 | 22.993 | 11.173 | 25.564 | 0.452 | 25.252 |
| 51 | 21 | 5 | 30 | 0 | 5.880 | 15.231 | 2.119 | 17.350 | 37.363 | 21.290 | 9.497 | 23.312 | 0.420 | 32.178 |
| 52 | 20 | 2 | 30 | 0 | 5.640 | 16.715 | 2.998 | 19.713 | 35.050 | 20.357 | 9.130 | 22.310 | 0.422 | 43.823 |
| 53 | 10 | 1 | 30 | 0 | 5.890 | 18.556 | 3.276 | 21.832 | 34.980 | 18.793 | 7.770 | 20.336 | 0.392 | 34.385 |
| 54 | 13 | 3 | 30 | 0 | 5.730 | 17.634 | 3.137 | 20.771 | 44.317 | 24.523 | 12.673 | 27.604 | 0.477 | 29.665 |
| 55 | 25 | 4 | 30 | 0 | 5.790 | 16.063 | 2.070 | 18.133 | 38.340 | 20.333 | 9.797 | 22.570 | 0.449 | 37.388 |
| 56 | 25 | 4 | 30 | 0 | 5.810 | 19.478 | 2.981 | 22.458 | 33.103 | 21.730 | 9.520 | 23.724 | 0.413 | 28.807 |
| 57 | 10 | 1 | 30 | 0 | 6.000 | 17.274 | 2.417 | 19.691 | 34.100 | 15.190 | . | . | . | 29.788 |
| 58 | 13 | 3 | 30 | 0 | 5.860 | 21.897 | 4.248 | 26.146 | 35.647 | 21.120 | 9.243 | 23.054 | 0.413 | 38.246 |
| 59 | 25 | 4 | 30 | 0 | . | 15.310 | 2.779 | 18.090 | 37.230 | 18.663 | 8.373 | 20.456 | 0.422 | 35.488 |
| 60 | 13 | 3 | 30 | 0 | 5.950 | 15.339 | 2.891 | 18.229 | 38.810 | 20.817 | 9.897 | 23.049 | 0.444 | 31.504 |
| 61 | 21 | 5 | 30 | 0 | . | 13.915 | 1.118 | 15.033 | . | . | . | . | . | 25.681 |
| 62 | 25 | 4 | 30 | 0 | 5.810 | 15.557 | 1.448 | 17.006 | 42.297 | 20.700 | 9.410 | 22.738 | 0.427 | 27.091 |
| 63 | 10 | 1 | 30 | 0 | . | 12.907 | 1.933 | 14.840 | 33.340 | 17.030 | 6.773 | 18.328 | 0.379 | 27.397 |
| 64 | 25 | 4 | 30 | 0 | 5.930 | 22.340 | . | 27.537 | 39.547 | 22.493 | 11.207 | 25.130 | 0.462 | 32.975 |
| 65 | 20 | 2 | 30 | 0 | 5.870 | 14.994 | 1.394 | 16.388 | 38.053 | 18.730 | 8.480 | 20.560 | 0.425 | . |
| 66 | 21 | 5 | 30 | 0 | 5.930 | 20.005 | 2.406 | 22.411 | 39.983 | 22.693 | 11.260 | 25.333 | 0.461 | 30.155 |
| 67 | 13 | 3 | 30 | 0 | . | 13.514 | 3.799 | 17.314 | . | . | . | . | . | 35.488 |
| 68 | 10 | 1 | 30 | 0 | 6.050 | 16.812 | 3.221 | 20.033 | 33.457 | 17.663 | 7.097 | 19.036 | 0.382 | 45.049 |
| 69 | 13 | 3 | 30 | 0 | 6.100 | . | . | . | 37.177 | 23.010 | 10.847 | 25.438 | 0.440 | 29.052 |
| 70 | 20 | 2 | 30 | 0 | . | 10.767 | 2.037 | 12.804 | 40.430 | 21.613 | 10.060 | 23.840 | 0.436 | 23.230 |
| 71 | 10 | 1 | 30 | 0 | 6.050 | 21.724 | 1.569 | 23.294 | 41.713 | 23.480 | 10.837 | 25.860 | 0.432 | 30.523 |
| 72 | 20 | 2 | 30 | 0 | 5.750 | 19.553 | 3.233 | 22.786 | 42.643 | 23.660 | 12.260 | 26.648 | 0.478 | 21.820 |
| 73 | 21 | 5 | 30 | 0 | 5.780 | 20.215 | 2.358 | 22.573 | 38.447 | 21.387 | 10.133 | 23.666 | 0.442 | 43.149 |
| 74 | 20 | 2 | 30 | 0 | 5.800 | 18.836 | 3.289 | 22.125 | 41.787 | 24.057 | 11.777 | 26.785 | 0.455 | 30.768 |
| 75 | 8 | 1 | 0 | 0 | 5.900 | 19.547 | 2.876 | 22.423 | 40.780 | 21.000 | 9.433 | 23.021 | 0.422 | 32.055 |
| 76 | 22 | 2 | 0 | 0 | 5.840 | 15.878 | 2.574 | 18.452 | 35.847 | 19.687 | 8.160 | 21.311 | 0.393 | 34.078 |
| 77 | 24 | 4 | 0 | 0 | 5.580 | 25.622 | 3.392 | 29.014 | 43.603 | 19.467 | 8.927 | 21.416 | 0.430 | 30.646 |
| 78 | 11 | 3 | 0 | 0 | 5.350 | 16.066 | 1.358 | 17.424 | 40.903 | 19.177 | 8.350 | 20.916 | 0.411 | 34.017 |
| 79 | 8 | 1 | 0 | 0 | 5.730 | 19.327 | 1.501 | 20.829 | 39.523 | 21.013 | 10.010 | 23.276 | 0.445 | 30.952 |
| 80 | 22 | 2 | 0 | 0 | 5.900 | 23.206 | 1.370 | 24.576 | 37.693 | 19.567 | 8.623 | 21.383 | 0.415 | 26.478 |
| 81 | 22 | 2 | 0 | 0 | . | 13.878 | 2.528 | 16.407 | 35.133 | 18.227 | 9.193 | 20.414 | 0.467 | 32.668 |
| 82 | 22 | 2 | 0 | 0 | 6.120 | 17.947 | 2.275 | 20.222 | 35.850 | 20.240 | 8.903 | 22.112 | 0.414 | 26.478 |
| 83 | 19 | 5 | 0 | 0 | 5.980 | 12.840 | 0.933 | 13.773 | 37.343 | 20.953 | 10.490 | 23.433 | 0.464 | 28.317 |
| 84 | 19 | 5 | 0 | 0 | 5.590 | 17.041 | 2.287 | 19.328 | 40.997 | 22.747 | 11.047 | 25.287 | 0.452 | 36.285 |
| 85 | 8 | 1 | 0 | 0 | 5.840 | 21.083 | 3.403 | 24.486 | 32.187 | 18.403 | 7.497 | 19.872 | 0.387 | . |
| 86 | 19 | 5 | 0 | 0 | 5.660 | 13.214 | 1.363 | 14.576 | 38.780 | 19.077 | 8.733 | 20.981 | 0.429 | 27.704 |
| 87 | 24 | 4 | 0 | 0 | 5.540 | 16.163 | 2.057 | 18.220 | 38.440 | 21.473 | 10.600 | 23.947 | 0.459 | 27.704 |
| 88 | 22 | 2 | 0 | 0 | . | 16.473 | 1.656 | 18.129 | 36.057 | 18.577 | 8.227 | 20.317 | 0.417 | 33.220 |
| 89 | 19 | 5 | 0 | 0 | 5.930 | 12.612 | . | 13.226 | 41.433 | 25.047 | 12.197 | 27.858 | 0.453 | 25.865 |
| 90 | 19 | 5 | 0 | 0 | 5.930 | 17.449 | 3.390 | 20.839 | 37.477 | 22.843 | 12.150 | 25.874 | 0.489 | 29.788 |
| 91 | 8 | 1 | 0 | 0 | 5.730 | 19.068 | 1.214 | 20.282 | 42.847 | 23.940 | 11.390 | 26.511 | 0.444 | 24.394 |
| 92 | 8 | 1 | 0 | 0 | . | 18.012 | 2.646 | 20.658 | 35.500 | 16.197 | 10.677 | 19.399 | 0.583 | 29.910 |
| 93 | 11 | 3 | 0 | 0 | 5.840 | 18.912 | 2.778 | 21.690 | 40.263 | 17.807 | 8.553 | 19.754 | 0.448 | 23.168 |
| 94 | 11 | 3 | 0 | 0 | 5.940 | . | 3.871 | . | 37.457 | 21.943 | 10.197 | 24.197 | 0.435 | 33.588 |
| 95 | 24 | 4 | 0 | 0 | 5.590 | 17.096 | 2.269 | 19.365 | 36.837 | 23.130 | 11.080 | 25.647 | 0.447 | 37.939 |
| 96 | 11 | 3 | 0 | 0 | 6.100 | 16.988 | 3.316 | 20.304 | 33.560 | 16.340 | 6.777 | 17.690 | 0.393 | 37.449 |
| 97 | 24 | 4 | 0 | 0 | 5.820 | 22.421 | 4.278 | 26.699 | 40.447 | 21.560 | 10.683 | 24.062 | 0.460 | 37.388 |
| 99 | 24 | 4 | 0 | 0 | 6.110 | 15.911 | 4.739 | 20.650 | 32.297 | 19.260 | 8.650 | 21.113 | 0.422 | . |
| 1 | 23 | 4 | 15 | 8 | . | . | . | . | 36.583 | 21.627 | 10.330 | 23.967 | 0.446 | 33.526 |
| 2 | 14 | 1 | 15 | 8 | 5.640 | 20.410 | 3.122 | 23.531 | 38.047 | 21.760 | 9.390 | 23.700 | 0.407 | 38.185 |
| 3 | 18 | 2 | 15 | 8 | 5.730 | 20.357 | 2.358 | 22.715 | 37.973 | 24.070 | 11.507 | 26.679 | 0.446 | 28.991 |
| 4 | 18 | 2 | 15 | 8 | 5.650 | 15.376 | 2.487 | 17.862 | 37.250 | 22.813 | 10.257 | 25.013 | 0.423 | . |
| 5 | 15 | 5 | 15 | 8 | 5.250 | 19.736 | . | 24.042 | 35.717 | 19.733 | 9.293 | 21.812 | 0.440 | 33.465 |
| 6 | 15 | 5 | 15 | 8 | 5.720 | 19.062 | 1.879 | 20.941 | 35.907 | 20.933 | 9.973 | 23.188 | 0.445 | 34.630 |
| 7 | 7 | 3 | 15 | 8 | 5.780 | 19.749 | 2.849 | 22.598 | 37.323 | 20.903 | 9.047 | 22.777 | 0.408 | 32.730 |
| 8 | 15 | 5 | 15 | 8 | 5.620 | 16.927 | 2.421 | 19.348 | 39.040 | 17.720 | 10.120 | 20.406 | 0.519 | 37.143 |
| 9 | 7 | 3 | 15 | 8 | 5.800 | 15.569 | 1.341 | 16.910 | 38.693 | 15.473 | 10.597 | 18.754 | 0.600 | 26.110 |
| 10 | 7 | 3 | 15 | 8 | 5.390 | 22.655 | 2.530 | 25.186 | 39.573 | 22.350 | 10.243 | 24.586 | 0.430 | 28.930 |
| 11 | 18 | 2 | 15 | 8 | 5.990 | 22.150 | 1.740 | 23.891 | 31.117 | 15.637 | . | . | . | 23.413 |
| 12 | 18 | 2 | 15 | 8 | 5.530 | 22.024 | . | . | 39.030 | 23.303 | 11.160 | 25.838 | 0.447 | . |
| 13 | 15 | 5 | 15 | 8 | 5.710 | 15.727 | 2.483 | 18.210 | 35.847 | 18.373 | 7.797 | 19.959 | 0.401 | 29.113 |
| 15 | 18 | 2 | 15 | 8 | 5.960 | 17.596 | 2.455 | 20.051 | 35.897 | 19.000 | 7.937 | 20.591 | 0.396 | 42.659 |
| 16 | 14 | 1 | 15 | 8 | 6.010 | 21.960 | 3.410 | 25.371 | 36.290 | 18.840 | 8.033 | 20.481 | 0.403 | 32.852 |
| 17 | 7 | 3 | 15 | 8 | 5.580 | 15.703 | 2.425 | 18.128 | 33.447 | 18.423 | 7.513 | 19.896 | 0.387 | 43.149 |
| 18 | 23 | 4 | 15 | 8 | 5.800 | 23.104 | 0.951 | 24.055 | 32.110 | 16.987 | 6.980 | 18.365 | 0.390 | . |
| 19 | 14 | 1 | 15 | 8 | 5.680 | 15.494 | 1.868 | 17.363 | 41.127 | 20.010 | 9.960 | 22.352 | 0.462 | 23.781 |
| 20 | 23 | 4 | 15 | 8 | 5.700 | 14.471 | 1.951 | 16.422 | 40.573 | 21.173 | 9.633 | 23.262 | 0.427 | 34.385 |
| 21 | 14 | 1 | 15 | 8 | 5.670 | 17.639 | 3.591 | 21.230 | 33.483 | 22.630 | 11.473 | 25.372 | 0.469 | 37.204 |
| 22 | 14 | 1 | 15 | 8 | 5.510 | 16.422 | 1.335 | 17.758 | 42.010 | 19.413 | 8.510 | 21.197 | 0.413 | 24.271 |
| 23 | 15 | 5 | 15 | 8 | 5.660 | 17.633 | 3.125 | 20.757 | 41.637 | 20.450 | 9.483 | 22.542 | 0.434 | 33.526 |
| 24 | 7 | 3 | 15 | 8 | 5.530 | 14.458 | 1.576 | 16.034 | 40.660 | 23.390 | 11.357 | 26.001 | 0.452 | 29.236 |
| 25 | 9 | 3 | 45 | 8 | 5.980 | 14.994 | 1.396 | 16.390 | 30.840 | 15.520 | 5.583 | 16.494 | 0.345 | 42.659 |
| 26 | 26 | 4 | 45 | 8 | 5.730 | 19.109 | 2.724 | 21.833 | 35.300 | 16.203 | 8.520 | 18.307 | 0.484 | 31.565 |
| 27 | 12 | 1 | 45 | 8 | 5.500 | 21.880 | 3.423 | 25.303 | 36.957 | 16.157 | 9.800 | 18.897 | 0.545 | 40.881 |
| 28 | 17 | 5 | 45 | 8 | 5.470 | 18.829 | 1.852 | 20.681 | 41.217 | 19.773 | 9.103 | 21.768 | 0.431 | 24.762 |
| 29 | 26 | 4 | 45 | 8 | 5.570 | 15.708 | 2.096 | 17.804 | 39.977 | 20.257 | 9.190 | 22.244 | 0.426 | 29.543 |
| 30 | 26 | 4 | 45 | 8 | 5.680 | 14.902 | 2.552 | 17.454 | . | 20.023 | 10.623 | 22.667 | 0.488 | 29.849 |
| 31 | 9 | 3 | 45 | 8 | 5.750 | 19.999 | 2.531 | 22.530 | 34.947 | 18.673 | 7.730 | 20.210 | 0.392 | 37.572 |
| 32 | 12 | 1 | 45 | 8 | 6.020 | 16.083 | 2.751 | 18.834 | 32.153 | 14.893 | . | . | . | 38.246 |
| 33 | 16 | 2 | 45 | 8 | 5.650 | 22.462 | 2.291 | 24.753 | 32.607 | 16.090 | 5.747 | 17.085 | 0.343 | . |
| 34 | 12 | 1 | 45 | 8 | 5.640 | 17.637 | 1.501 | 19.138 | 37.487 | 20.823 | 8.503 | 22.493 | 0.388 | 38.001 |
| 35 | 17 | 5 | 45 | 8 | 5.790 | 16.283 | 1.393 | 17.676 | 39.400 | 21.727 | 8.940 | 23.494 | 0.390 | 23.536 |
| 36 | 26 | 4 | 45 | 8 | 5.870 | 18.984 | 2.093 | 21.077 | . | 17.260 | 8.527 | 19.251 | 0.459 | 42.843 |
| 37 | 16 | 2 | 45 | 8 | 5.720 | 15.600 | 1.304 | 16.904 | 41.183 | . | 11.987 | . | . | 25.191 |
| 38 | 16 | 2 | 45 | 8 | 5.850 | 17.917 | 2.750 | 20.667 | 37.343 | 20.213 | 9.400 | 22.292 | 0.435 | 21.146 |
| 39 | 9 | 3 | 45 | 8 | 5.400 | 20.436 | 2.119 | 22.555 | 37.957 | 20.790 | 8.990 | 22.650 | 0.408 | 31.014 |
| 40 | 12 | 1 | 45 | 8 | 5.830 | 16.078 | 2.392 | 18.470 | 39.307 | 20.473 | 8.997 | 22.363 | 0.414 | 26.355 |
| 41 | 17 | 5 | 45 | 8 | 5.210 | 17.386 | 2.421 | 19.807 | 39.423 | 23.980 | 11.447 | 26.572 | 0.445 | 29.788 |
| 42 | 17 | 5 | 45 | 8 | 5.800 | 16.248 | 3.004 | 19.252 | 36.700 | 18.900 | 9.533 | 21.168 | 0.467 | 36.407 |
| 43 | 16 | 2 | 45 | 8 | 5.990 | 17.779 | 1.713 | 19.493 | 37.360 | 18.823 | 8.037 | 20.467 | 0.404 | 25.559 |
| 44 | 9 | 3 | 45 | 8 | 5.710 | 21.632 | 1.378 | 23.010 | 39.237 | 20.367 | 9.683 | 22.551 | 0.444 | 23.965 |
| 45 | 9 | 3 | 45 | 8 | 5.350 | . | 3.184 | . | 38.917 | 20.783 | 9.907 | 23.024 | 0.445 | 33.649 |
| 46 | 17 | 5 | 45 | 8 | 5.650 | 17.454 | 1.365 | 18.819 | 38.790 | 20.130 | 8.747 | 21.948 | 0.410 | 28.439 |
| 47 | 12 | 1 | 45 | 8 | 5.260 | 15.992 | 1.445 | 17.437 | 39.277 | 19.860 | 8.823 | 21.732 | 0.418 | 21.759 |
| 48 | 26 | 4 | 45 | 8 | 5.490 | 21.276 | 2.123 | 23.399 | 38.140 | 22.073 | 10.197 | 24.315 | 0.433 | 33.526 |
| 49 | 16 | 2 | 45 | 8 | . | 13.604 | . | 13.920 | . | 14.717 | . | . | . | 21.575 |
| 50 | 21 | 5 | 30 | 8 | 5.640 | 12.548 | 1.457 | 14.005 | 37.830 | 16.657 | 9.640 | 19.245 | 0.525 | 26.846 |
| 51 | 21 | 5 | 30 | 8 | 5.870 | 16.330 | 2.143 | 18.473 | 33.733 | 18.373 | 7.227 | 19.743 | 0.375 | 35.120 |
| 52 | 20 | 2 | 30 | 8 | 5.880 | 14.770 | 2.373 | . | 36.233 | 19.840 | 8.427 | 21.555 | 0.402 | 35.365 |
| 53 | 10 | 1 | 30 | 8 | 5.660 | 22.925 | . | 29.172 | 34.247 | 19.833 | 8.627 | 21.628 | 0.410 | 38.798 |
| 54 | 13 | 3 | 30 | 8 | 5.780 | 13.341 | 1.751 | 15.093 | 38.103 | . | 9.407 | . | . | 21.526 |
| 55 | 25 | 4 | 30 | 8 | 5.740 | 14.047 | 2.339 | 16.386 | 35.947 | 18.507 | 8.543 | 20.383 | 0.432 | 40.452 |
| 56 | 25 | 4 | 30 | 8 | 5.760 | 16.379 | . | 20.667 | 33.547 | 16.080 | 8.280 | 18.087 | 0.476 | 26.907 |
| 57 | 10 | 1 | 30 | 8 | 6.010 | 15.225 | 1.708 | 16.933 | 32.687 | . | . | . | . | 40.391 |
| 58 | 13 | 3 | 30 | 8 | 5.930 | 16.514 | 3.172 | 19.686 | 34.307 | 18.223 | 7.277 | 19.622 | 0.380 | 35.304 |
| 59 | 25 | 4 | 30 | 8 | 5.590 | 14.662 | 1.486 | 16.148 | 38.063 | 20.133 | 8.793 | 21.970 | 0.412 | 26.233 |
| 60 | 13 | 3 | 30 | 8 | 5.800 | 16.141 | 3.078 | 19.219 | 39.683 | 19.507 | 8.583 | 21.312 | 0.415 | 26.417 |
| 61 | 21 | 5 | 30 | 8 | . | 13.127 | 1.074 | 14.201 | . | . | . | . | . | 31.136 |
| 62 | 25 | 4 | 30 | 8 | 5.590 | 16.822 | 2.539 | 19.362 | 40.937 | 21.337 | 10.297 | 23.691 | 0.450 | 26.601 |
| 63 | 10 | 1 | 30 | 8 | 5.530 | 19.491 | 2.306 | 21.798 | 31.357 | 22.267 | 10.587 | 24.655 | 0.444 | 41.311 |
| 64 | 25 | 4 | 30 | 8 | 5.700 | 12.629 | 2.322 | 14.951 | 35.353 | 22.027 | 9.830 | 24.121 | 0.420 | 29.788 |
| 65 | 20 | 2 | 30 | 8 | 5.720 | 21.174 | 1.701 | 22.875 | 39.850 | 19.407 | 9.057 | 21.416 | 0.437 | 43.211 |
| 66 | 21 | 5 | 30 | 8 | 5.720 | 16.229 | 2.814 | 19.043 | 38.023 | 19.430 | 8.573 | 21.237 | 0.416 | 26.049 |
| 67 | 13 | 3 | 30 | 8 | . | 10.483 | 1.533 | . | 30.247 | . | . | . | . | 21.207 |
| 68 | 10 | 1 | 30 | 8 | 5.990 | 20.393 | 3.535 | 23.928 | 33.523 | 17.917 | 7.483 | 19.417 | 0.396 | 31.872 |
| 69 | 13 | 3 | 30 | 8 | 5.500 | 13.541 | 1.367 | 14.908 | 38.910 | 22.250 | 10.617 | 24.653 | 0.445 | 19.675 |
| 70 | 20 | 2 | 30 | 8 | 5.810 | 18.481 | 2.368 | 20.849 | 37.257 | 23.077 | 11.003 | 25.566 | 0.445 | 37.081 |
| 71 | 10 | 1 | 30 | 8 | 6.050 | 20.055 | 2.104 | 22.159 | 42.443 | 20.077 | 9.777 | 22.331 | 0.453 | 33.343 |
| 72 | 20 | 2 | 30 | 8 | 5.400 | 17.184 | 2.881 | 20.064 | 43.023 | . | 12.207 | . | . | 28.072 |
| 73 | 21 | 5 | 30 | 8 | 5.750 | 14.969 | 1.853 | 16.822 | 38.247 | 21.387 | 9.700 | 23.484 | 0.426 | 31.749 |
| 74 | 20 | 2 | 30 | 8 | 5.680 | 21.569 | 3.783 | 25.352 | 37.053 | 21.727 | 10.360 | 24.070 | 0.445 | 45.049 |
| 75 | 8 | 1 | 0 | 8 | 5.780 | 20.525 | 2.302 | 22.827 | 42.597 | 20.927 | 10.287 | 23.318 | 0.457 | 27.091 |
| 76 | 22 | 2 | 0 | 8 | 5.960 | 19.112 | 2.918 | 22.029 | 34.900 | 20.510 | 9.057 | 22.421 | 0.416 | 36.223 |
| 77 | 24 | 4 | 0 | 8 | 5.720 | 14.348 | 1.271 | 15.620 | 42.277 | 16.513 | 7.857 | 18.287 | 0.444 | 21.391 |
| 78 | 11 | 3 | 0 | 8 | 5.690 | . | 0.941 | 24.618 | 40.853 | 19.743 | 9.810 | 22.046 | 0.461 | 26.907 |
| 79 | 8 | 1 | 0 | 8 | 5.780 | 22.045 | 2.582 | 24.627 | 34.670 | 18.963 | 8.320 | 20.708 | 0.413 | 33.649 |
| 80 | 22 | 2 | 0 | 8 | 5.970 | 19.695 | 2.611 | 22.305 | 32.180 | 16.783 | 6.977 | 18.176 | 0.394 | 39.656 |
| 81 | 22 | 2 | 0 | 8 | 5.790 | 19.655 | 2.945 | 22.600 | 31.897 | 15.987 | 7.287 | 17.569 | 0.428 | 41.249 |
| 82 | 22 | 2 | 0 | 8 | 5.760 | 18.029 | 0.959 | 18.989 | 35.480 | 19.023 | 8.007 | 20.640 | 0.398 | 21.207 |
| 83 | 19 | 5 | 0 | 8 | 5.690 | 14.414 | 2.150 | 16.563 | 42.250 | 22.480 | 10.703 | 24.898 | 0.444 | 23.413 |
| 84 | 19 | 5 | 0 | 8 | 5.860 | 18.342 | 1.294 | 19.636 | 41.783 | 21.183 | 9.477 | 23.206 | 0.421 | 24.517 |
| 85 | 8 | 1 | 0 | 8 | 5.560 | . | . | . | 36.027 | 19.957 | 8.573 | 21.720 | 0.406 | . |
| 86 | 19 | 5 | 0 | 8 | 5.590 | . | . | . | 38.697 | 20.723 | 10.167 | 23.083 | 0.456 | . |
| 87 | 24 | 4 | 0 | 8 | 5.680 | 17.406 | 2.265 | 19.670 | 39.737 | 22.660 | 10.627 | 25.028 | 0.439 | 22.188 |
| 88 | 22 | 2 | 0 | 8 | 5.960 | 16.769 | 1.149 | 17.918 | 35.603 | 16.680 | 6.157 | 17.780 | 0.354 | 26.539 |
| 89 | 19 | 5 | 0 | 8 | 5.650 | 11.852 | 0.752 | . | 42.167 | 22.520 | 11.133 | 25.122 | 0.459 | 25.191 |
| 90 | 19 | 5 | 0 | 8 | 5.550 | 12.004 | 1.776 | 13.780 | 36.273 | 23.277 | 10.910 | 25.707 | 0.438 | 21.955 |
| 91 | 8 | 1 | 0 | 8 | 5.600 | 17.274 | 3.542 | 20.816 | 40.710 | 23.080 | 10.843 | 25.500 | 0.439 | 30.707 |
| 92 | 8 | 1 | 0 | 8 | 5.770 | 20.713 | 1.370 | 22.084 | 39.357 | 22.790 | 9.837 | 24.822 | 0.407 | 29.923 |
| 93 | 11 | 3 | 0 | 8 | 5.210 | 19.436 | 2.455 | 21.891 | . | 21.167 | 10.360 | 23.566 | 0.455 | 25.375 |
| 94 | 11 | 3 | 0 | 8 | 5.880 | 19.463 | 2.627 | 22.090 | 37.807 | 17.513 | 9.213 | 19.789 | 0.484 | 32.975 |
| 95 | 24 | 4 | 0 | 8 | 5.570 | 14.785 | 1.676 | 16.461 | 37.527 | 21.260 | 9.623 | 23.337 | 0.425 | 32.607 |
| 96 | 11 | 3 | 0 | 8 | 5.950 | 15.563 | 2.984 | 18.547 | 32.910 | 16.290 | . | . | . | . |
| 97 | 24 | 4 | 0 | 8 | 5.710 | 14.683 | 1.244 | 15.927 | 39.737 | 16.090 | 9.390 | 18.630 | 0.528 | 28.807 |
| 99 | 24 | 4 | 0 | 8 | 5.920 | 16.616 | 2.273 | 18.889 | 33.383 | 18.857 | 7.647 | 20.348 | 0.385 | 38.552 |
| 1 | 23 | 4 | 15 | 16 | 5.620 | 16.747 | 2.711 | 19.457 | 39.383 | 21.787 | 9.640 | 23.824 | 0.417 | 32.546 |
| 2 | 14 | 1 | 15 | 16 | 5.450 | 19.718 | 1.325 | 21.043 | 40.193 | 19.370 | 7.947 | 20.937 | 0.389 | 31.075 |
| 3 | 18 | 2 | 15 | 16 | 5.680 | 17.087 | 1.609 | 18.696 | 42.283 | 23.207 | 10.143 | 25.327 | 0.412 | 23.965 |
| 4 | 18 | 2 | 15 | 16 | 5.880 | 16.306 | 1.421 | 17.728 | 37.547 | 20.120 | 8.513 | 21.847 | 0.400 | 28.501 |
| 5 | 15 | 5 | 15 | 16 | 5.730 | 15.934 | 1.663 | 17.598 | 40.073 | . | 11.250 | . | . | 22.310 |
| 6 | 15 | 5 | 15 | 16 | 5.720 | 17.453 | 2.522 | 19.975 | 36.037 | 21.163 | 9.673 | 23.269 | 0.429 | 34.201 |
| 7 | 7 | 3 | 15 | 16 | 5.920 | 16.849 | 2.836 | 19.684 | 37.163 | 21.143 | 9.307 | 23.101 | 0.415 | 29.297 |
| 8 | 15 | 5 | 15 | 16 | 5.600 | 15.565 | 2.227 | 17.792 | 38.640 | 18.057 | 10.053 | 20.667 | 0.508 | 28.930 |
| 9 | 7 | 3 | 15 | 16 | 5.860 | 17.165 | 2.721 | 19.885 | 42.917 | 18.370 | 10.170 | 20.997 | 0.506 | 28.317 |
| 10 | 7 | 3 | 15 | 16 | 5.430 | 21.980 | . | . | 41.247 | 22.580 | 10.943 | 25.092 | 0.451 | 30.094 |
| 11 | 18 | 2 | 15 | 16 | 5.410 | 16.797 | 2.378 | 19.176 | 37.930 | 23.467 | 10.947 | 25.894 | 0.436 | 20.471 |
| 12 | 18 | 2 | 15 | 16 | 6.000 | 14.648 | 1.500 | 16.148 | 34.680 | 16.653 | . | . | . | 30.094 |
| 13 | 15 | 5 | 15 | 16 | 5.780 | 15.702 | 2.181 | 17.883 | 40.167 | 23.493 | 11.057 | 25.965 | 0.440 | 22.617 |
| 15 | 18 | 2 | 15 | 16 | 6.020 | . | 1.202 | . | 33.687 | 17.593 | 7.227 | 19.020 | 0.390 | 22.494 |
| 16 | 14 | 1 | 15 | 16 | 5.890 | . | 2.574 | 25.539 | 34.673 | 17.707 | 7.087 | 19.072 | 0.381 | . |
| 17 | 7 | 3 | 15 | 16 | 5.830 | 17.046 | 1.790 | 18.836 | 36.783 | 18.937 | 7.897 | 20.517 | 0.395 | 37.388 |
| 18 | 23 | 4 | 15 | 16 | 5.680 | 19.323 | 3.482 | 22.805 | 34.297 | 18.270 | 7.227 | 19.647 | 0.377 | . |
| 19 | 14 | 1 | 15 | 16 | 5.400 | 19.090 | 1.755 | 20.845 | 41.010 | 17.343 | 8.657 | 19.384 | 0.463 | 25.007 |
| 20 | 23 | 4 | 15 | 16 | 5.690 | 13.566 | . | 14.087 | 40.437 | 20.710 | 9.297 | 22.701 | 0.422 | 29.113 |
| 21 | 14 | 1 | 15 | 16 | 5.700 | 19.656 | . | 23.719 | 37.810 | 19.987 | 9.263 | 22.029 | 0.434 | 37.572 |
| 22 | 14 | 1 | 15 | 16 | 5.840 | 13.440 | 2.230 | 15.670 | 42.817 | 21.770 | 10.057 | 23.981 | 0.433 | 24.946 |
| 23 | 15 | 5 | 15 | 16 | 5.780 | 15.605 | 1.856 | 17.461 | 42.887 | 20.040 | 9.460 | 22.161 | 0.441 | 26.208 |
| 24 | 7 | 3 | 15 | 16 | 5.710 | 16.855 | 1.774 | 18.630 | 36.993 | 20.483 | 8.727 | 22.265 | 0.403 | 29.420 |
| 25 | 9 | 3 | 45 | 16 | 5.910 | 19.614 | 2.799 | 22.413 | 32.830 | 18.720 | 7.800 | 20.280 | 0.395 | 34.078 |
| 26 | 26 | 4 | 45 | 16 | 5.680 | 14.427 | 1.884 | 16.310 | 40.053 | 23.173 | 10.390 | 25.396 | 0.421 | 23.597 |
| 27 | 12 | 1 | 45 | 16 | 5.680 | 19.124 | 2.516 | 21.640 | 39.920 | 21.347 | 9.283 | 23.278 | 0.410 | 32.055 |
| 28 | 17 | 5 | 45 | 16 | 5.730 | . | 2.473 | . | 44.390 | 21.273 | 10.183 | 23.585 | 0.446 | 31.994 |
| 29 | 26 | 4 | 45 | 16 | 5.730 | 16.275 | 2.581 | 18.857 | . | 23.253 | 10.567 | 25.542 | 0.427 | 34.446 |
| 30 | 26 | 4 | 45 | 16 | 5.650 | 17.861 | 1.517 | 19.377 | . | 20.767 | 10.093 | 23.090 | 0.452 | 28.255 |
| 31 | 9 | 3 | 45 | 16 | 5.700 | 16.303 | . | 15.961 | 36.787 | 20.300 | 8.973 | 22.195 | 0.416 | 25.130 |
| 32 | 12 | 1 | 45 | 16 | . | 16.281 | 1.658 | 17.939 | . | 16.120 | . | . | . | 35.304 |
| 33 | 16 | 2 | 45 | 16 | 5.780 | 20.646 | 1.803 | 22.449 | 35.360 | 16.913 | 6.783 | 18.223 | 0.381 | 41.127 |
| 34 | 12 | 1 | 45 | 16 | 5.800 | 19.150 | 1.010 | 20.160 | 39.650 | 23.000 | 9.767 | 24.988 | 0.402 | 38.491 |
| 35 | 17 | 5 | 45 | 16 | . | 13.166 | 1.538 | 14.704 | 39.593 | 21.050 | 8.583 | 22.733 | 0.387 | 30.707 |
| 36 | 26 | 4 | 45 | 16 | 5.780 | 15.402 | 2.346 | 17.748 | . | 18.167 | 7.073 | 19.495 | 0.371 | 21.329 |
| 37 | 16 | 2 | 45 | 16 | 5.610 | 17.390 | 1.852 | 19.243 | 39.977 | 20.513 | 9.710 | 22.695 | 0.442 | 29.910 |
| 38 | 16 | 2 | 45 | 16 | 5.770 | 17.482 | . | 21.411 | 41.720 | 22.187 | 10.440 | 24.520 | 0.440 | 26.294 |
| 39 | 9 | 3 | 45 | 16 | 5.800 | 17.129 | 2.372 | 19.501 | 38.773 | 23.430 | 10.867 | 25.827 | 0.434 | 25.375 |
| 40 | 12 | 1 | 45 | 16 | 5.830 | 14.614 | 2.031 | 16.645 | 39.450 | 20.680 | 8.987 | 22.548 | 0.410 | 33.097 |
| 41 | 17 | 5 | 45 | 16 | 5.640 | 14.606 | 3.243 | 17.849 | 36.313 | . | 10.330 | . | . | 26.355 |
| 42 | 17 | 5 | 45 | 16 | 5.780 | 15.063 | 1.947 | 17.010 | 36.770 | 23.983 | 10.897 | 26.343 | 0.426 | 29.297 |
| 43 | 16 | 2 | 45 | 16 | 5.710 | . | . | . | 38.200 | 18.233 | 8.880 | 20.281 | 0.453 | 31.075 |
| 44 | 9 | 3 | 45 | 16 | 5.690 | 16.932 | 2.868 | 19.800 | 42.293 | 22.063 | 11.153 | 24.722 | 0.468 | 37.081 |
| 45 | 9 | 3 | 45 | 16 | 5.730 | 22.670 | 2.339 | 25.009 | 40.870 | 23.590 | 10.400 | 25.781 | 0.415 | 23.965 |
| 46 | 17 | 5 | 45 | 16 | 5.280 | . | . | . | 38.003 | . | 7.253 | . | . | 26.784 |
| 47 | 12 | 1 | 45 | 16 | 5.740 | 14.129 | 1.398 | 15.527 | 42.567 | 23.353 | 10.930 | 25.785 | 0.438 | . |
| 48 | 26 | 4 | 45 | 16 | 5.680 | 17.027 | 1.628 | 18.654 | 40.880 | 21.323 | 9.127 | 23.194 | 0.404 | 28.439 |
| 49 | 16 | 2 | 45 | 16 | . | 14.941 | 1.033 | 15.974 | . | 16.083 | . | . | . | 26.968 |
| 50 | 21 | 5 | 30 | 16 | 5.620 | 13.090 | 1.534 | 14.624 | 41.487 | 23.943 | 11.477 | 26.552 | 0.447 | 26.294 |
| 51 | 21 | 5 | 30 | 16 | 5.800 | 16.333 | 1.659 | 17.992 | 35.090 | 17.850 | 7.023 | 19.182 | 0.375 | 18.326 |
| 52 | 20 | 2 | 30 | 16 | 5.870 | 13.718 | 1.238 | 14.957 | 36.823 | 20.390 | 9.180 | 22.361 | 0.423 | 31.504 |
| 53 | 10 | 1 | 30 | 16 | 5.760 | 20.558 | 2.083 | 22.641 | 35.600 | 21.927 | 9.840 | 24.033 | 0.422 | 35.672 |
| 54 | 13 | 3 | 30 | 16 | 5.790 | 18.805 | 1.402 | 20.207 | 41.010 | 22.860 | 10.843 | 25.301 | 0.443 | 33.588 |
| 55 | 25 | 4 | 30 | 16 | 5.680 | 20.048 | 2.788 | 22.836 | 39.567 | 20.980 | 10.317 | 23.379 | 0.457 | 24.455 |
| 56 | 25 | 4 | 30 | 16 | 5.790 | 17.894 | 3.042 | 20.936 | 36.280 | 22.247 | 9.683 | 24.263 | 0.411 | 32.914 |
| 57 | 10 | 1 | 30 | 16 | 5.900 | 16.493 | 2.075 | 18.568 | 34.090 | 17.363 | 6.167 | 18.426 | 0.341 | 25.436 |
| 58 | 13 | 3 | 30 | 16 | 5.740 | 15.919 | 2.813 | 18.732 | 36.633 | 20.050 | 8.577 | 21.807 | 0.404 | 38.491 |
| 59 | 25 | 4 | 30 | 16 | 5.660 | 16.018 | 2.155 | 18.173 | 37.113 | 18.853 | 9.610 | 21.161 | 0.471 | 27.017 |
| 60 | 13 | 3 | 30 | 16 | 5.780 | 18.431 | 2.459 | 20.889 | 34.843 | 15.977 | . | . | . | 29.175 |
| 61 | 21 | 5 | 30 | 16 | . | 14.593 | 1.771 | 16.363 | . | 15.307 | . | . | . | 26.478 |
| 62 | 25 | 4 | 30 | 16 | 5.680 | 20.204 | 1.800 | 22.004 | 42.750 | 20.737 | 9.250 | 22.706 | 0.420 | 30.339 |
| 63 | 10 | 1 | 30 | 16 | 5.720 | 14.433 | 2.227 | 16.660 | 34.897 | 19.440 | 9.520 | 21.646 | 0.455 | 34.078 |
| 64 | 25 | 4 | 30 | 16 | 5.760 | 13.635 | 1.944 | 15.580 | 36.793 | 20.610 | 8.967 | 22.476 | 0.410 | 34.385 |
| 65 | 20 | 2 | 30 | 16 | 5.600 | 15.612 | 1.786 | 17.398 | 38.730 | 20.117 | 9.327 | 22.174 | 0.434 | 30.584 |
| 66 | 21 | 5 | 30 | 16 | 5.530 | 18.259 | 1.651 | 19.910 | 39.713 | 22.317 | 10.437 | 24.637 | 0.437 | 36.162 |
| 67 | 13 | 3 | 30 | 16 | . | 12.613 | 2.444 | 15.057 | . | . | 6.267 | . | . | 23.720 |
| 68 | 10 | 1 | 30 | 16 | 5.700 | 19.551 | 2.741 | 22.291 | 34.347 | 18.207 | 6.987 | 19.501 | 0.366 | . |
| 69 | 13 | 3 | 30 | 16 | 5.830 | 15.378 | 2.047 | 17.425 | 37.537 | 18.790 | 9.427 | 21.022 | 0.465 | . |
| 70 | 20 | 2 | 30 | 16 | 5.780 | 19.909 | 1.962 | 21.871 | 36.143 | 21.703 | 9.847 | 23.833 | 0.426 | 22.126 |
| 71 | 10 | 1 | 30 | 16 | 5.640 | 17.302 | 2.376 | 19.678 | 42.420 | 18.770 | 8.370 | 20.552 | 0.419 | 28.317 |
| 72 | 20 | 2 | 30 | 16 | 5.810 | . | 2.854 | 14.152 | 41.520 | 24.963 | 12.250 | 27.807 | 0.456 | 23.475 |
| 73 | 21 | 5 | 30 | 16 | 5.780 | 15.557 | 1.717 | 17.273 | 40.423 | 23.023 | 10.790 | 25.426 | 0.438 | . |
| 74 | 20 | 2 | 30 | 16 | 5.730 | 19.009 | 2.785 | 21.794 | 40.767 | 24.873 | 11.343 | 27.338 | 0.428 | 34.752 |
| 75 | 8 | 1 | 0 | 16 | 5.640 | . | 2.377 | . | 42.460 | 18.573 | 8.433 | 20.398 | 0.426 | 32.975 |
| 76 | 22 | 2 | 0 | 16 | 5.700 | 15.687 | 2.450 | 18.137 | 36.587 | 21.113 | 8.690 | 22.832 | 0.390 | 26.417 |
| 77 | 24 | 4 | 0 | 16 | 5.820 | 16.806 | 2.582 | 19.388 | 42.907 | 19.510 | 8.800 | 21.403 | 0.424 | 26.907 |
| 78 | 11 | 3 | 0 | 16 | 5.820 | 16.882 | 2.039 | 18.922 | 40.413 | 18.000 | 7.883 | 19.651 | 0.413 | 21.084 |
| 79 | 8 | 1 | 0 | 16 | 5.740 | 17.423 | 1.344 | 18.767 | 38.687 | 19.287 | 8.640 | 21.134 | 0.421 | 28.378 |
| 80 | 22 | 2 | 0 | 16 | 5.530 | 14.120 | 0.687 | 14.807 | 35.183 | 17.747 | 6.793 | 19.002 | 0.366 | 31.749 |
| 81 | 22 | 2 | 0 | 16 | 5.840 | 19.852 | 2.473 | 22.325 | 35.907 | 17.667 | 7.250 | 19.096 | 0.389 | 32.239 |
| 82 | 22 | 2 | 0 | 16 | 5.780 | 21.657 | 1.433 | 23.089 | 39.523 | 20.687 | 8.987 | 22.554 | 0.410 | 25.252 |
| 83 | 19 | 5 | 0 | 16 | 5.660 | 16.700 | . | 21.026 | 42.637 | 21.040 | 9.040 | 22.900 | 0.406 | 31.688 |
| 84 | 19 | 5 | 0 | 16 | 5.750 | 17.388 | 3.209 | 20.596 | 40.820 | 21.117 | 9.073 | 22.983 | 0.406 | 28.010 |
| 85 | 8 | 1 | 0 | 16 | 5.650 | 16.502 | 1.673 | 18.175 | 36.090 | 19.183 | 7.780 | 20.701 | 0.385 | . |
| 86 | 19 | 5 | 0 | 16 | 5.690 | 14.585 | 1.448 | 16.033 | 40.697 | 23.060 | 10.430 | 25.309 | 0.425 | 22.739 |
| 87 | 24 | 4 | 0 | 16 | 5.860 | 18.777 | 2.291 | 21.068 | 39.787 | 22.043 | 9.850 | 24.144 | 0.420 | 30.155 |
| 88 | 22 | 2 | 0 | 16 | 6.060 | 16.983 | 1.414 | 18.398 | 36.207 | 16.553 | . | . | . | 36.039 |
| 89 | 19 | 5 | 0 | 16 | 5.210 | 18.902 | . | 23.310 | 41.227 | 21.023 | 11.040 | 23.746 | 0.484 | 25.375 |
| 90 | 19 | 5 | 0 | 16 | 5.770 | 16.569 | 1.862 | 18.430 | 38.650 | . | . | . | . | 23.781 |
| 91 | 8 | 1 | 0 | 16 | 5.580 | 21.924 | 2.032 | 23.956 | 38.797 | 19.513 | 8.960 | 21.472 | 0.430 | 23.904 |
| 92 | 8 | 1 | 0 | 16 | 5.920 | 17.980 | 1.806 | 19.786 | 38.457 | 22.707 | 10.093 | 24.849 | 0.418 | 24.333 |
| 93 | 11 | 3 | 0 | 16 | 5.480 | 15.554 | 1.802 | 17.356 | . | 21.400 | 10.573 | 23.870 | 0.459 | 21.697 |
| 94 | 11 | 3 | 0 | 16 | 5.740 | 18.337 | 2.660 | 20.998 | 37.897 | 22.180 | 9.900 | 24.289 | 0.420 | 36.468 |
| 95 | 24 | 4 | 0 | 16 | 5.640 | 18.531 | 2.249 | 20.780 | 37.907 | 22.290 | 9.900 | 24.390 | 0.418 | 27.152 |
| 96 | 11 | 3 | 0 | 16 | 5.950 | 15.492 | 1.347 | 16.839 | 39.733 | 20.237 | 8.527 | 21.960 | 0.399 | . |
| 97 | 24 | 4 | 0 | 16 | 5.820 | 19.347 | 1.645 | 20.993 | 42.894 | 21.497 | 9.953 | 23.689 | 0.434 | 26.723 |
| 99 | 24 | 4 | 0 | 16 | 5.830 | 16.976 | 2.836 | 19.812 | 37.193 | 20.847 | 9.707 | 22.996 | 0.436 | 35.304 |
