# Supplementary material for: The inclusion of de-oiled wet distillers grains in feedlot diets reduces the expression of lipogenic genes and fat content in Longissimus muscle from F1 Angus-Nellore cattle
Source: PeerJ. 2019 Oct 28;7:e7699. doi: 10.7717/peerj.7699 (PMC6822641; doi:10.7717/peerj.7699)
Supplement: Data S3 [file peerj-07-7699-s003.docx]

| Animal | Treatment | Block | Pen | SCD1 | PPARy | SRBP1 | ACOX | FAS | CPT2 | FABP4 | GPX1 | LPL | PPARa | ACACA |
| --- | --- | --- | --- | --- | --- | --- | --- | --- | --- | --- | --- | --- | --- | --- |
| 1 | 15 | 2 | 23 | 0.867 | 0.110 | 0.370 | 0.540 | 0.304 | 0.109 | 2.228 | 0.883 | 0.752 | 0.758 | 1.099 |
| 6 | 15 | 2 | 15 | 0.363 | 0.096 | 0.415 | 0.356 | 0.185 | 0.182 | 0.190 | 0.584 | 0.385 | 0.304 | 0.795 |
| 7 | 15 | 1 | 7 | 0.566 | 0.078 | 0.311 | 0.399 | 0.219 | 0.146 | 0.512 | 0.411 | 0.471 | 0.848 | 0.657 |
| 9 | 15 | 1 | 7 | 0.289 | 0.132 | 0.686 | 0.723 | 0.107 | 0.043 | 0.140 | 1.256 | 1.507 | 1.495 | 0.648 |
| 10 | 15 | 1 | 7 | 0.144 | 0.048 | 0.384 | 0.387 | 0.189 | 0.198 | 0.078 | 0.609 | 0.754 | 1.004 | 0.607 |
| 12 | 15 | 1 | 18 | 0.558 | 0.188 | 0.548 | 0.529 | 0.548 | 0.178 | 0.626 | 0.763 | 0.495 | 0.108 | 0.063 |
| 13 | 15 | 2 | 15 | 0.334 | 0.110 | 0.617 | 0.593 | 0.323 | 0.209 | 0.145 | 0.497 | 0.618 | 0.141 | 0.114 |
| 20 | 15 | 2 | 23 | 0.378 | 0.089 | 1.439 | 0.470 | 0.082 | 0.037 | 0.433 | 1.055 | 0.533 | 2.161 | 1.260 |
| 23 | 15 | 2 | 15 | 0.180 | 0.869 | 3.732 | 0.135 | 0.396 | 0.060 | 0.125 | 0.683 | 0.492 | 3.154 | 1.952 |
| 24 | 15 | 1 | 7 | 0.356 | 0.125 | 1.820 | 0.837 | 0.188 | 0.040 | 0.324 | 1.612 | 0.743 | 6.445 | 3.615 |
| 25 | 45 | 1 | 9 | 0.443 | 0.118 | 0.376 | 0.436 | 0.242 | 0.179 | 1.456 | 0.968 | 0.146 | 0.462 | 0.444 |
| 31 | 45 | 1 | 9 | 0.372 | 0.256 | 0.705 | 0.350 | 0.218 | 0.042 | 0.555 | 0.512 | 0.993 | 1.094 | 0.409 |
| 32 | 45 | 1 | 12 | 0.106 | 0.052 | 0.402 | 0.364 | 0.139 | 0.095 | 0.114 | 0.290 | 0.348 | 0.707 | 0.746 |
| 33 | 45 | 1 | 16 | 0.209 | 0.039 | 0.486 | 0.457 | 0.213 | 0.116 | 0.609 | 0.490 | 0.572 | 0.336 | 0.437 |
| 35 | 45 | 2 | 17 | 0.522 | 0.047 | 0.461 | 0.517 | 0.229 | 0.182 | 0.388 | 0.688 | 0.354 | 0.410 | 0.400 |
| 37 | 45 | 1 | 16 | 0.310 | 0.171 | 1.310 | 0.649 | 0.174 | 0.064 | 0.568 | 0.931 | 1.718 | 1.957 | 1.001 |
| 41 | 45 | 2 | 17 | 0.401 | 0.119 | 1.344 | 0.943 | 0.151 | 0.045 | 0.097 | 1.459 | 1.902 | 2.018 | 0.853 |
| 46 | 45 | 2 | 17 | 0.866 | 0.092 | 0.520 | 0.832 | 0.346 | 0.389 | 0.323 | 0.652 | 1.759 | 0.322 | 0.389 |
| 47 | 45 | 1 | 12 | 0.607 | 0.084 | 0.659 | 0.614 | 0.362 | 0.135 | 1.970 | 0.859 | 0.581 | 2.597 | 3.263 |
| 48 | 45 | 2 | 26 | 0.806 | 0.140 | 1.149 | 0.968 | 0.306 | 0.224 | 0.321 | 1.115 | 0.923 | 7.507 | 6.419 |
| 55 | 30 | 2 | 25 | 1.939 | 0.802 | 1.468 | 1.009 | 1.091 | 0.908 | 1.006 | 1.458 | 1.640 | 7.404 | 6.818 |
| 60 | 30 | 1 | 13 | 0.771 | 0.108 | 0.500 | 0.757 | 0.197 | 0.267 | 0.639 | 0.944 | 0.790 | 2.049 | 1.869 |
| 61 | 30 | 2 | 21 | 1.142 | 0.131 | 1.142 | 1.288 | 0.476 | 0.298 | 0.981 | 1.679 | 0.888 | 0.496 | 0.498 |
| 62 | 30 | 2 | 25 | 3.789 | 0.190 | 2.493 | 2.309 | 0.846 | 0.431 | 0.927 | 1.513 | 1.258 | 4.871 | 5.216 |
| 67 | 30 | 1 | 13 | 0.133 | 0.056 | 0.921 | 0.363 | 0.142 | 0.041 | 0.100 | 1.346 | 0.929 | 1.688 | 0.776 |
| 70 | 30 | 1 | 20 | 0.641 | 0.074 | 0.715 | 0.484 | 0.221 | 0.231 | 0.111 | 0.440 | 0.383 | 0.495 | 0.270 |
| 71 | 30 | 1 | 10 | 1.437 | 0.218 | 1.661 | 1.366 | 0.361 | 0.417 | 2.569 | 0.860 | 1.439 | 1.325 | 0.499 |
| 73 | 30 | 2 | 21 | 0.345 | 1.587 | 0.510 | 0.441 | 1.739 | 1.480 | 0.501 | 0.543 | 0.904 | 3.308 | 1.910 |
| 78 | 0 | 1 | 11 | 1.234 | 1.723 | 1.824 | 2.282 | 1.472 | 0.968 | 0.600 | 1.069 | 1.634 | 2.082 | 1.486 |
| 79 | 0 | 1 | 8 | 0.876 | 0.319 | 0.598 | 0.572 | 0.459 | 0.310 | 1.513 | 0.622 | 0.234 | 0.033 | 0.038 |
| 82 | 0 | 1 | 22 | 1.290 | 0.166 | 1.331 | 0.949 | 0.456 | 2.582 | 1.523 | 1.637 | 0.649 | 0.270 | 0.557 |
| 84 | 0 | 2 | 19 | 1.445 | 2.012 | 1.056 | 1.413 | 1.506 | 1.392 | 0.470 | 1.591 | 1.822 | 2.864 | 2.197 |
| 92 | 0 | 1 | 8 | 0.966 | 1.270 | 1.198 | 1.614 | 1.460 | 1.252 | 0.577 | 1.178 | 1.287 | 0.060 | 0.041 |
| 95 | 0 | 2 | 24 | 0.909 | 0.132 | 0.508 | 0.891 | 0.344 | 0.331 | 0.416 | 0.687 | 0.613 | 0.099 | 0.205 |
| 96 | 0 | 1 | 11 | 0.366 | 1.178 | 0.829 | 0.411 | 1.482 | 1.154 | 0.569 | 0.742 | 0.801 | 1.433 | 2.469 |
| 99 | 0 | 2 | 24 | 0.742 | 0.749 | 0.940 | 0.587 | 0.711 | 0.390 | 2.991 | 0.962 | 0.948 | 1.291 | 1.814 |
